# Competition for Dominance Within Replicating Quasispecies During Prolonged SARS-CoV-2 Infection in an Immunocompromised Host

**DOI:** 10.1093/ve/veac042

**Published:** 2022-05-21

**Authors:** Francesca Caccuri, Serena Messali, Daria Bortolotti, Dario Di Silvestre, Antonella De Palma, Chiara Cattaneo, Anna Bertelli, Alberto Zani, Maria Milanesi, Marta Giovanetti, Giovanni Campisi, Valentina Gentili, Antonella Bugatti, Federica Filippini, Erika Scaltriti, Stefano Pongolini, Alessandra Tucci, Simona Fiorentini, Pasqualina d’Ursi, Massimo Ciccozzi, Pierluigi Mauri, Roberta Rizzo, Arnaldo Caruso

**Affiliations:** Section of Microbiology Department of Molecular and Translational Medicine, University of Brescia, 25123 Brescia, Italy; Section of Microbiology Department of Molecular and Translational Medicine, University of Brescia, 25123 Brescia, Italy; Department of Chemical, Pharmaceutical and Agricultural Sciences, University of Ferrara, Ferrara, Italy; Proteomic and Metabolomic Laboratory, Institute of Biomedical Technologies, National Research Council (ITB-CNR), 20054 Segrate, Italy; Proteomic and Metabolomic Laboratory, Institute of Biomedical Technologies, National Research Council (ITB-CNR), 20054 Segrate, Italy; Department of Hematology, ASST Spedali Civili di Brescia, Brescia, Italy; Section of Microbiology Department of Molecular and Translational Medicine, University of Brescia, 25123 Brescia, Italy; Section of Microbiology Department of Molecular and Translational Medicine, University of Brescia, 25123 Brescia, Italy; Section of Experimental Oncology and Immunology, Department of Molecular and Translational Medicine, University of Brescia, 25123 Brescia, Italy; Laboratório de Flavivírus, Instituto Oswaldo Cruz, Fundação Oswaldo Cruz, Rio de Janeiro, Brazil; Laboratório de Genética Celular e Molecular, Instituto de Ciências Biológicas, Universidade Federal de Minas Gerais, Belo Horizonte, Minas Gerais, Brazil; Section of Microbiology Department of Molecular and Translational Medicine, University of Brescia, 25123 Brescia, Italy; Department of Chemical, Pharmaceutical and Agricultural Sciences, University of Ferrara, Ferrara, Italy; Section of Microbiology Department of Molecular and Translational Medicine, University of Brescia, 25123 Brescia, Italy; Section of Microbiology Department of Molecular and Translational Medicine, University of Brescia, 25123 Brescia, Italy; Risk Analysis and Genomic Epidemiology Unit, Istituto Zooprofilattico Sperimentale della Lombardia e dell’Emilia-Romagna, 43126 Parma, Italy; Risk Analysis and Genomic Epidemiology Unit, Istituto Zooprofilattico Sperimentale della Lombardia e dell’Emilia-Romagna, 43126 Parma, Italy; Department of Hematology, ASST Spedali Civili di Brescia, Brescia, Italy; Section of Microbiology Department of Molecular and Translational Medicine, University of Brescia, 25123 Brescia, Italy; Institute of Technologies in Biomedicine, National Research Council, 20090 Segrate, Italy; Unit of Medical Statistics and Molecular Epidemiology, University Campus Bio-Medico of Rome, Rome, Italy; Proteomic and Metabolomic Laboratory, Institute of Biomedical Technologies, National Research Council (ITB-CNR), 20054 Segrate, Italy; Department of Chemical, Pharmaceutical and Agricultural Sciences, University of Ferrara, Ferrara, Italy; Section of Microbiology Department of Molecular and Translational Medicine, University of Brescia, 25123 Brescia, Italy

**Keywords:** SARS-CoV-2 Quasispecies, prolonged infection, Viral competition, Immunocompromised host, Innate immunity, RNA sensing, Interferons, Proteomic analysis

## Abstract

SARS-CoV-2 variants of concern (VOCs) emerge for their capability to better adapt to the human host aimed and enhance human-to-human transmission. Mutations in spike largely contributed to adaptation. Viral persistence is a prerequisite for intra-host virus evolution, and this likely occurred in immunocompromised patients who allow intra-host long-term viral replication. The underlying mechanism leading to the emergence of variants during viral persistence in the immunocompromised host is still unknown. Here we show the existence of an ensemble of minor mutants in the early biological samples obtained from an immunocompromised patient and their dynamic interplay with the master mutant during a persistent and productive long-term infection. In particular, after 222 days of active viral replication the original master mutant, named MB61°, was replaced by a minor quasispecies (MB61^222^) expressing two critical mutations in spike, namely Q493K and N501T. Isolation of the two viruses allowed us to show that MB61^222^ entry into target cells occurred mainly by the fusion at the plasma membrane (PM), whereas endocytosis characterized the entry mechanism used by MB61°. Interestingly, co-infection of two human cell lines of different origin with the SARS-CoV-2 isolates highlighted the early and dramatic predominance of MB61^222^ over MB61° replication. This finding may be explained by a faster replicative activity of MB61^222^ as compared to MB61° as well as by the capability of MB61^222^ to induce peculiar viral RNA sensing mechanisms leading to an increased production of Interferons (IFNs) and, in particular, of IFN-induced transmembrane protein 1 (IFITM1) and IFITM2. Indeed, it has been recently shown that IFITM2 is able to restrict SARS-CoV-2 entry occurring by endocytosis. In this regard, MB61^222^ may escape the antiviral activity of IFITMs by using the PM fusion pathway for entry into the target cell, whereas MB61° cannot escape this host antiviral response during MB61^222^ co-infection, since it has endocytosis as main pathway of entry. Altogether, our data support the evidence of quasispecies fighting for host dominance by taking benefit from the cell machinery to restrict the productive infection of competitors in the viral ensemble. This finding may explain, at least in part, the extraordinary rapid worldwide turnover of VOCs that use the PM fusion pathway to enter into target cells over the original pandemic strain.

## 1. Introduction

Severe Acute Respiratory Syndrome Coronavirus 2 (SARS-CoV-2) emerged in December 2019 in Wuhan, China, and then spread worldwide causing the coronavirus-disease 19 (COVID-19) pandemic (Alteri et al., 2021). In the general population, clinical features of COVID-19 range from asymptomatic to critical illness. However, adult patients with hematologic malignancies and COVID-19 have a worse outcome and high risk of dying (Passamonti et al., 2020; Vijenthira et al., 2020). In October 2020, the United Kingdom faced a rapid rise in positive COVID-19 cases sustained by a new single phylogenetic cluster that led to the identification of a new SARS-CoV-2 lineage, namely Alpha (B.1.1.7), promptly defined as a variant of concern (VOC) (Challen et al., 2021; European Centre for Disease Prevention and Control (ECDC), 2020). Soon after, in South Africa, Brazil, and India, new circulating lineages also defined VOCs were described and designated Beta (B.1.351) (Tegalli et al., 2021), Gamma (P.1) (Faria et al, 2021), and Delta (B.1.617.2) (Dhar et al., 2021). In November 2021 the last VOC named Omicron (B.1.1.529) emerged in South Africa (Viana et al., 2022) and it is actually start replacing all other VOCs worldwide and dominating the pandemic scenario.

All VOCs are characterized by a typical mutational pattern in the spike receptor binding domain (RBD), the most variable part of the coronavirus genome (Wu et al., 2020; Zhou et al., 2020), leading to human angiotensin-converting enzyme 2 (ACE2) increased affinity and consequently, enhanced infectivity (Starr et al, 2020; Nelson et al, 2021). This evidence suggests that viral evolution is ongoing and aimed to adapt to the host and acquire more fitness.

Viral persistence is a prerequisite for intra-host virus evolution (Kang et al., 2020; Zapor, 2020). Duration of SARS-CoV-2 shedding through respiratory secretions is highly variable among individuals and is independent from the presence of symptoms (Lee et al., 2020), ranging from few days to more than one month (Cevik et al., 2021). Prolonged detection of viral RNA genome and

SARS-CoV-2 positivity have been documented (Choi et al. 2020; Kang et al., 2020) but it is increasingly evident that viral transmission posed by post-convalescent COVID-19 patients may be negligible. For example, in one study of healthcare workers self-isolating due to persistent RT-PCR positivity up to 55 days after the onset of symptoms, no viable virus was recoverable in 29 out of 29 nasopharyngeal/oropharyngeal samples tested (Laferl et al., 2021). However, there is no evidence whether these results can be applied to special populations such as immunocompromised patients. Recent reports have, in fact, described cases where the virus can be isolated more than two months after its first detection (Avanzato et al., 2020; Choi et al. 2020).

The reasons why some people have productive long-term infection have been not yet completely understood even if this phenomenon has been found to occur in immunocompromised patients only (Camprubí et al., 2020; O'Sullivan et al., 2020; Abdul-Jawad et al., 2021; Baang et al., 2021; Hensley et al., 2021; Siqueira et al., 2021). It has been suggested that the emergence of VOCs worldwide has likely occurred in immunocompromised patients who allowed intra-host persistent infection and variations in viral population (Al Khatib et al., 2020; Avanzato et al., 2020; Choi et al., 2020; Kemp et al., 2020; Lythgoe et al., 2021; Wang et al., 2021a; Wang et al., 2021b; Valesano et al., 2021). Indeed, phylogenetic, populational and computational analyses of viral sequences showed that increased diversity was associated with prolonged viral replication (Voloch et al., 2021). It is worth noting that the RNA virus population in a host is not represented by a single dominating sequence, but rather consists of an ensemble of replicating viruses characterized by closely related sequences termed quasispecies (Sun et al., 2021). This allows RNA viruses to have a greater possibility to find the best adapting quasispecies to the specific host, usually achieved by changes in functional genes such as the S gene (Zhou et al., 2020). These findings raise the question about the underlying mechanism leading to the emergence of variants during viral persistence in the immunocompromised host.

Here we describe the case of an immunocompromised patient affected by B-cell lymphoma where the persistent replication of SARS-CoV-2 led to the accumulation of critical amino acid (aa)

changes within the spike protein RBD. These mutations occurred early during infection and reflected the dynamic changes of quasispecies through competitive interactions in the viral population, probably aimed to determine a better replicative activity and overcome selective immune responses. Our results provide new insights into the intra-host evolution highlighting competition among replicating ensembles for dominance.

## 2. Materials and methods

### 2.1 Specimen collection

From April 2nd to December 28th, 2020, nasopharyngeal swabs (FLOQSwabs COPAN, Brescia, Italy) were periodically collected in universal transport medium (UTM, COPAN) for diagnostic purposes during hospitalization or at the Brescia community testing site. Samples were transported to the Microbiology Division of the Brescia Civic Hospital (Brescia, Italy). This work was approved by the Brescia Ethical Committee and the patient provided written consent to publish this case study.

### 2.2 SARS-CoV-2 detection in nasopharyngeal samples

Nasopharyngeal samples were processed using the InGenius automatic system (ElitechGroup, Turin, Italy), according to the manufacturer's instructions. The presence of SARS-CoV-2 nucleic acid was evaluated using the GeneFinder™ COVID-19 PLUS RealAmp Kit (Osang Healthcare, Anyang-si, Gyeonggi-do, Republic of Korea) whose reagents detect conserved regions in ORF1ab, E and N genes of the SARS-CoV-2 genome. Cycle threshold (Ct) values and results were automatically calculated by InGenius analysis software.

### 2.3 Cells

Vero E6 and Caco-2 cell lines were obtained from Istituto Zooprofilattico Sperimentale (Brescia, Italy) and maintained in Dulbecco's Modified Eagle Medium (DMEM; Gibco, Thermo Fisher Scientific, Waltham, MA, USA) supplemented with 10% fetal bovine serum (FBS; Gibco, Thermo Fisher Scientific).

Calu-3 cell line (HTB-55) was purchased from the American Type Cell Culture Collection (ATCC, Rockville, MD, USA). Cells were cultured in complete Eagle's minimum essential medium (MEM; Gibco, Thermo Fisher Scientific) supplemented with 10% FBS, 1% Sodium Pyruvate (Gibco,

Thermo Fisher Scientific), and 1% L-glutamine (Gibco, Thermo Fisher Scientific). Cells were maintained at 37°C in a humidified atmosphere of 5% $CO_2$.

### 2.4 Viral infection

Infection experiments were run as previously described (Caccuri et al., 2020; Caruso et al., 2020) using the clinical SARS-CoV-2 isolates $MB61^0$ and $MB61^{222}$ (Fiorentini et al, 2021). The viruses were propagated in Vero E6 cells and the viral titer was determined by a standard plaque assay. All the experiments were performed in a biosafety level-3 (BLS-3) laboratory using viruses at a Multiplicity of Infection (MOI) of 0.01. When reported, the cells were infected simultaneously with both $MB61^0$ and $MB61^{222}$ SARS-CoV-2 isolates at a MOI of 0.01 each.

### 2.5 Growth kinetics of SARS-CoV-2 isolates

Calu-3 and Caco-2 cells were infected in culture media without FBS containing $MB61^0$ and $MB61^{222}$ SARS-CoV-2 isolates at passage 1, alone or in combination, soon after having confirmed their identity with the original patient's sample by next generation whole-genome sequencing. After a 1 h incubation at 37°C and 5% $CO_2$, the inoculum was removed, cells were washed twice with phosphate buffered saline (PBS) and replaced with 3 ml of culture media supplemented with 2% FBS. Supernatant samples were taken at 0, 12, 24, 48, 72, and 96 h post infection (p.i.). Release of viable virus was evaluated by standard plaque assay and digital droplets polymerase chain reaction (ddPCR).

### 2.6 ddPCR

Total RNA was extracted using the QIAamp DSP Virus kit (Qiagen, Hilden, Germany) following manufacturer's guidelines. DdPCR reaction, was carried out on a QX200 ddPCR system with Automated Droplet Generator (Bio-Rad Laboratories, Hercules, CA, USA) using the One-Step SARS-CoV-2 ddPCR Kit (Bio-Rad Laboratories). Fluorescence of each droplet was evaluated using the QX 200 Droplet Reader (Bio-Rad Laboratories) equipped with QuantaSoft v1.7 Software. To evaluate the viral load in ddPCR we used the QuantaSoft Analysis Pro v1.0 Software (Bio-Rad Laboratories) that accompanied the droplet reader to calculate the concentration of the target sequences, along with Poisson-based 95% confidence intervals. The positive populations for each probe are identified using SARS-CoV-2 Standard positive control supplied with the One-Step SARS-CoV-2 ddPCR Kit (Bio-Rad Laboratories). The declared analytic sensitivity of the ddPCR kit is 0.260 cp/µl to 0.351 cp/µl for N1 and N2 probe sets, respectively. The data generated by the QX 200 droplet reader were rejected from subsequent analysis if a low number of total droplets was measured in 22 µl PCR (<10,000), or if all of the droplets were positive (saturation of the reaction) (Racki et al., 2014).

**2.7 Illumina sequencing**

An amplicon-based approach, targeting 343 nucleotide-long partially overlapping subgenomic regions that cover the entire SARS-CoV-2 genome, was used. Total RNA was extracted from 200 µl UTM. Full-length SARS-CoV-2 genomes were then generated using Paragon Genomics' CleanPlex multiplex PCR Research and Surveillance Panel, according to the manufacturer's protocol (Al Khabit et al., 2020; Li et al., 2020; Alteri et al. 2021). Purified library was quantified with Qubit Fluorometer (Qubit DNA HS Assay Kit, Thermo Fisher Scientific). Amplicon library was loaded in a 300-cycle sequencing cartridge and the deep sequencing was performed on MiSeq platform (Illumina, San Diego, CA, USA). Raw data were checked for quality using FastQC (https://www.bioinformatics.babraham.ac.uk/projects/fastqc/) and then analyzed with the specifically designed software SOPHiA GENETICS' SARS-CoV-2 Panel (SOPHiA GENETICS, Lausanne, Switzerland). To confirmed data analyses, the paired-end reads were trimmed with Trimmomatic ver. 0.38 for quality (Q score > 25) and length (> 36 bp) and then were analyzed with Geneious® software (version 11.1.5) (Biomatters Ltd, Auckland, New Zealand). Consensus sequence was reconstructed by mapping the reads to the SARS-CoV-2 reference sequence

NC_045512 using Bowtie2 in sensitive-local mode with consensus threshold at 65%. The variant calling was carried out by the Variant Finder Tool (Geneious) filtering out variants with a p value greater than 0, using a minimum variant frequency of 0 and default parameters for Maximum Variant p-value ($10^{-6}$). To minimize false discoveries, a stringent approach to evaluated the presence of quasispecies spectrum in the patient's samples was applied. An intra-host single nucleotide variant (iSNV) was identified at genome positions with > 4000-fold sequencing coverage and with at least four reads supporting the nucleotide substitution. Amino acid substitutions at the identified iSNV sites were measured as the proportion of paired-end mapped reads with the alternative amino acid.

**2.8 Phylogenetic analysis**

We combined the 12 sequential viral consensus genomes obtained from the patient with 2419 sequences available on GISAID (https://www.gisaid.org/) and representative of the SARS-CoV-2 lineage B.1.1 globally circulating until November 2021. Only genomes > 29,000 bp and < 1% of ambiguities were retrieved, low-quality genomes (> 10% of ambiguous positions), were excluded. Sequences were aligned using MAFFT (FF-NS-2 algorithm) employing default parameters (Nakamura et al., 2018). The alignment was manually curated to optimize number and location of gaps using Aliview (Larsson et al, 2014). Lineage assessment was conducted using the Phylogenetic Assignment of Named Global Outbreak LINeages tool available at https://github.com/hCoV-2019/pangolin (O'Toole et al., 2020). Phylogenetic analysis was performed using the maximum likelihood (ML) method implemented in IQ-TREE2 employing the GTR+I model of nucleotide substitution (Minh et al., 2020). The statistical robustness of individual nodes was determined using the SH-aLTR branch support. The branches in the ML-tree were then converted into units of calendar time in TreeTime (Sagulenko et al., 2018) using a constant rate of 0.0008 substitutions/site/year (Wilkinson et al., 2021) with a clock standard deviation of 0.0004 substitutions/site/year.

## 2.9 Molecular modelling

The starting structure for the wild type hACE2 protein in complex with the SARS-CoV-2 spike protein RBD (PDB ID 6M0J) was obtained from the RCSB Protein Data Bank. Homology modeling SWISS-MODEL web server was used both for adding missing residue and constructing Q493K-N501T model structures (Waterhouse et al., 2018). The Zinc ion was preserved from the crystal structure. Molecular dynamics simulations (MDs) of the two complexes (WT, Q493K-N501T) were performed using Amber 18 (Case et al., 2018) package and the ff14SB force field parameters was used for protein. Each complex was solvated in a periodic cubic water box using the TIP3P water model with 12 Å between the solutes and the edges of the box, and then a suitable number of $Na^+$ and $Cl^-$ ions was added to neutralize the whole systems. Each system was energy minimized with 500 steps of steepest descent followed by 500 steps of conjugate gradient, with positional restraints of 2 kcal/mol $A^2$ on all protein atoms, with a cut-off for non-bonded interactions of 8 Å, this minimization protocol was done four time. The systems were then subjected to heating procedure of 25000 steps, from 0.1 to 300 K in an NVT ensemble with a Langevin thermostat. Bonds involving hydrogen atoms were constrained with the SHAKE algorithm and 2 fs time step was used. The systems were then equilibrated at 300 K in 2 consecutive steps of 500 ps each in the NPT. In the second equilibration step the positional restraints were removed. MDs were performed over 200 ns using the pmemd CUDA program of the Amber18 package and a server Tesla K20 Graphical Processing Unit. The trajectories were analyzed to compare and observe the structural deviation between WT and mutant structure. The conformational ensembles along the last 50 ns of the trajectory were clustered by CPPTRAJ using hierarchical agglomerative algorithm. Hydrogen bonds analysis were carried out using CPPTRAJ obtaining the hydrogen bond frequency for each residue pair during the molecular dynamics. In order to identify salt bridge and VdW interactions of each representative structure PBePISA web server (Krissinel and Henrick, 2007) and RING2.0 web server (Piovesan et al., 2016) were used.

Electrostatic potential maps for the WT and mutant structures were calculated using the Adaptive Poisson Boltzmann Solver (APBS) (Jurrus et al, 2018).

**2.10 Fusion at the plasma membrane (PM) and endocytosis inhibition evaluation**

Endocytosis was inhibited by incubation with 25 μM Chlorpromazine (Sigma-Aldrich, Saint Louis, MO, USA). Cathepsin-mediated proteolysis was prevented by the addition of 20 μM Cathepsin L inhibitor III (Calbiochem; Sigma-Aldrich). PM fusion was inhibited by treating with 100 μM Camostat Mesylate (Sigma-Aldrich). Calu-3 cell pretreatment was performed by incubating for 1 h at 37°C. Then, media were removed and wells were washed twice with PBS. Untreated and treated cells were then infected. Viral titration was determined by Real Time qPCR at 24 and 48 h p.i., as follows. RNA extraction was performed with MagMAX Viral/Pathogen Nuclei Acid Isolation kit (Thermo Fisher Scientific) as previously described (Bortolotti, D., et al., 2020). SARS-CoV-2 titration was obtained by TaqMan 2019nCoV assay kit v1 RealTime-qPCR (Thermo Fisher Scientific).

**2.11 Gene Expression Analysis**

RNA sensors pathway genes, cytokines, interferons, IFITMs and LYE6 expression was evaluated on RNA extracted by using the RNeasy kit (Qiagen). DNase treatment was used to check for contaminant DNA presence, using β-actin PCR as a control. RT2 first strand kit (Qiagen) was used for RNA reverse transcription and cDNAs were immediately used or stored at -20°C. Gene expression analysis was performed by real-time quantitative PCR using PowerUp SYBR Green Master Mix (Thermo Fisher Scientific) and RIG-I (Hs.PT.58.4273674), TLR3 (Hs.PT.58.25887499.g), TLR7 (Hs.PT.58.39183219.g), IRF3 (Hs.PT.58.27933933.g), NF-κB (Hs.PT.58.20344216), IL-1α (Hs.PT.58.40913627), IL-1 (Hs.PT.58.1518186), IL-4 (Hs.PT.58.46539563.g), Il-6 (Hs.PT.58.40226675), TNF-α (Hs.PT.58.45380900), IFN-α (Hs.PT.58.24294810.g), IFN-β (Hs.PT.58.39481063.g), IFN-γ (Hs.PT.58.3781960), IFTIM1

(Hs.PT.58.25215635), IFITM2 (Hs.PT.58.1885104.g), IFITM3 (Hs.PT.58.40706345.g), LY6E (Hs.PT.58.22888825.g) or GAPDH (Hs.PT.58.25887499.g) PrimeTime qPCR primer assays (Integrated DNA Technologies IDT, Leuven, Belgium).

**2.12 RNA Sensor pathways analysis**

The evaluation of RNA sensors expression was performed using specific agonists: RIG-I/MDA5 agonist 5' triphosphate hairpin RNA complexed with transfection reagent Lyovec (1 µg/ml) (Invivogen, San Diego, CA, USA); TLR3 agonist Poly (I:C) (HMW) (2 µg/ml) (Invivogen); TLR7/8 Agonist - Imidazoquinoline compound R848 (2 µg/ml) (Invivogen).

**2.13 IRF3, NF-κB Expression and Phosphorylation Analysis**

The evaluation of IRF3 and NFκB expression and phosphorylation status was performed using the detection kit human total IRF-3 and phospho-IRF-3 (S386) ELISA kit (RayBiotech, Peachtree Corners, GA, USA), and total NF-κB p65 and phospho-NF-κB p65 (S536) (Abcam, Cambridge, UK) on cell lysates.

**2.14 Western Blot analysis**

RIG-I, TLR3 and TLR7 protein expression were quantified by Western blot assay. Whole cell lysates were treated with RIPA buffer containing proteinase inhibitor cocktail (Sigma-Aldrich). Total proteins were biotinylated with 0.2 mg/ml EZ-Linc Sulfo-NHS-LC-Biotin (Pierce, Rockford, IL, USA), immunoprecipitated for 90 min at 4°C with specific mAbs (anti-RIG-I, Clone OTI6C1; anti-TLR3, Clone 27N3D4; anti-TLR7, Clone NBP2-24905, and anti- β actin, Clone NB600-501) (Novus Biologics, Centennial, CO, USA), and incubated overnight at 4 °C with protein-G Sepharose beads (Santa Cruz Biotechnology, Dallas, TX, USA). The samples were resuspended in 20 µl Laemmli buffer (Bio-Rad). Twenty µg of immunoprecipitated proteins were loaded in each well and evaluated in denaturating conditions in 10% TGX-Pre-cast gel (Bio-Rad), with subsequent electroblotting transfer onto a PVDF membrane (Millipore, Burlington, MA, USA). The membrane was incubated with horseradish peroxidase (HRP)-conjugated streptavidin (Amersham Biosciences, Piscataway, NJ, USA) and developed with the ECL kit (Amersham Biosciences). The images were acquired by Geliance 600 (Perkin Elmer, Waltham, MA, USA).

**2.15 Quantitative ELISA**

IL-1 α, IL-1 β, IL-4, IL-6, IFN- α, IFN-β, and INF-γ levels were evaluated in cell culture supernatants by single ELISA kit assays (myBio-source, San Diego, CA, USA) following the customer's protocols.

**2.16 Protein extraction and enzymatic digestion**

The proteomic analysis described in this study was performed on not infected (NI) Calu-3 cells and on Calu-3 cells infected with SARS-CoV-2 $MB61^{0}$ and $MB61^{222}$ isolates, collected at 12, 24 and 48 h p.i.. Cells were lysed and proteins were extracted, reduced/alkylated and enzymatically digested using Easy Pep™ Mini MS Sample Prep Kit (Thermo Fisher Scientific). Tryptic and Lys-C digestion was carried out on 80 µg of extracted proteins mixture. Following the kit protocol, peptides were generated, cleaned-up to prepare detergent-free samples and resuspended in 0.1% formic acid (Sigma-Aldrich) for LC-MS/MS analysis in less than 3 h for each examined condition

**2.17 LC-MS/MS Analysis**

Peptide mixtures were analyzed using Eksigent nanoLC-Ultra® 2D System (Eksigent, part of AB SCIEX Dublin, CA, USA) combined with cHiPLC-nanoflex system (Eksigent) in trap-elute mode. Briefly, for each condition three technical replicates were performed, injecting 0.8 µg of proteins on the cHiPLC trap (200 µm x 500 µm ChromXP C18-CL, 3 µm, 120 Å, Eksigent, part of AB SCIEX Dublin, CA, USA) and running the loading pump in isocratic mode with 0.1% formic acid in water for 10 min at a flow of 3 µL/min. The automatic switching of cHiPLC ten-port valve then eluted the trapped mixture on a nano cHiPLC column (75 µm x 15 cm ChromXP C18-CL, 3 µm, 120 Å, Eksigent, part of AB SCIEX Dublin, CA, USA) through a 132 min gradient of eluent B (eluent A, 0.1% formic acid in water; eluent B, 0.1% formic acid in acetonitrile) at a flow rate of 300 nL/min. In depth, gradient was: from 5-10% B in 3 min, 10-35% B in 107 min, 35-95% B in 10 min and holding at 95% B for 12 min.

The eluted peptides were directly analyzed on an Orbitrap Exploris 120 mass spectrometer (Thermo Fisher Scientific) equipped with EASY-Spray ion source (Thermo Fisher Scientific). Easy spray was achieved using an EASY-Spray Emitter (Thermo Fisher Scientific) (nanoflow 7 µm ID Transfer Line 20 µm x 50 cm) held to 1.6 kV, while the ion transfer capillary was held at 220°C. Data-dependent acquisition (DDA) was performed, acquiring precursor ions in m/z range of 375–1,500 with a resolution (at m/z 200) of 60,000 FWHM (full width at half maximum). Precursor fragmentation was carried out at a resolution (at *m/z* 200) of 15,000 FWHM, using higher-energy collisional dissociation (HCD) method with normalized collision energy (NCE) of 30 eV and a dynamic exclusion of 20 sec. MS and MS/MS data were acquired in profile and centroid mode, respectively, using positive polarity and isotope exclusion was on. Isolation width was set at 2 m/z and first mass was set to 120 m/z. Mass spectrometer scan functions and high-performance liquid chromatography solvent gradients were controlled by the Xcalibur data system version 4.4 (Thermo Fisher Scientific) and Eksigent Control Software version 4.3 (Eksigent, part of AB SCIEX Dublin, CA, USA), respectively.

**2.18 Data Handling**

All data generated were searched using the Sequest HT search engine contained in Proteome Discoverer software, version 2.1 (Thermo Fisher Scientific). Experimental MS/MS spectra were compared with the theoretical mass spectra obtained by *in silico* digestion of 29 SARS-CoV-2 protein sequences obtained from Uniprot (www.uniprot.org) and Homo Sapiens proteome database (74842 entries), downloaded on March 2021. The following criteria were used for the identification of peptide sequences and related proteins: trypsin and Lys-C as enzymes, methionine oxidation, carbamidomethyl cysteine, three missed cleavages per peptide, mass tolerances of ± 10 ppm for precursor ions and ± 0.05 Da for fragment ions. Percolator node was used with a target-decoy strategy to give a final false discovery rates (FDR) at Peptide Spectrum Match (PSM) level of 0.01 (strict) based on q-values, considering maximum deltaCN of 0.05 (Käll et al., 2007). Only peptides with minimum peptide length of six amino acids, and rank 1 were considered. Protein grouping and strict parsimony principle were applied. Results were then exported to Excel file for further processing.

### 2.19 Differential expression and network analysis

The 42 LC/MS lists (6 runs for the 7 available conditions) obtained from the Proteome Discoverer software were aligned, normalized using a total signal normalization method (Griffin et al., 2010) and compared using a label-free quantification approach based on aPSMs, as previously reported (Caccuri et al., 2021). Data matrix dimensionality of 5025 distinct proteins was reduced by linear discriminant analysis (LDA). Following this procedure, we selected and retained proteins with $P<0.0001$, corresponding to a LDA F ratio > 11. Differentially expressed proteins (DEPs) selected by LDA were processed by Hierarchical Clustering applying the *Ward*'s method and the *Euclidean* distance metric. All processing was performed by JMP15.2 SAS software. Starting from DEPs selected by LDA, pairwise comparisons of cells, infected by the two virus strains and collected at different times p.i., were performed by Dave and DCI indices (Di Silvestre et al., 2013) and a *Homo sapiens* protein-protein interaction (PPI) network model was reconstructed by STRING Cytoscape's App (Doncheva et al., 2019). Protein-protein interactions were filtered by considering exclusively those "experiments" or/and "databases" annotated, with a STRING Score ≥ 0.15 and ≥ 0.35, respectively. Reconstructed networks were visualized and analyzed at functional level by Cytoscape v.3.8.2 and its apps (Su et al., 2014). In particular, DEPs were grouped in functional modules by the support of the GO enrichment tool inserted in STRING (Doncheva et al., 2019) and BINGO (Maere et al., 2005) Cytoscape's apps.

**2.20 Statistical Analysis.**

Data were analyzed for statistical significance using the Student's unpaired two-tailed t-test or two-way ANOVA when appropriate. The Bonferroni post-test was used to compare data. Differences were considered significant when $p < 0.05$. Statistical tests were performed using Prism 8 software (GraphPad Software, La Jolla, CA, USA).

## 3. Results

### 3.1 Clinical case

On March 25, 2020, a 59-year-old man with a 24-year history of follicular lymphoma was admitted to the hospital for lymphadenopathies, declining edema and light breathing difficulties. The presence of a bilateral pleural effusion, a segmental pulmonary embolism and a minimal pericardial effusion suggested a transformation of follicular lymphoma toward an aggressive non-Hodgkin lymphoma (NHL). Due to a subsequent development of a bilateral interstitial pneumonia and the presence of a hypoxemic respiratory failure, on April 2, 2020 (day 0) the patient was subjected to nasopharyngeal swab for SARS-CoV-2 testing and was first diagnosed positive for infection. COVID-19-related patient's symptoms required oxygen therapy administered concomitantly with intravenous methylprednisolone and the standard thromboprophylaxis dose of low molecular weight heparin. On day 8 the bone marrow biopsy confirmed the presence of a high-grade large B-cell lymphoma (95% of the bone marrow cells) and, upon the hematological diagnosis, the patient started CHOP (cyclophosphamide, doxorubicin, vincristine, and prednisone) chemotherapy associated with the B cell depleting monoclonal antibody Rituximab (R-CHOP). R-CHOP cycles were well tolerated and administered till day 53, when he was discharged with no more evident signs of COVID-19. In fact, at that time, he was eupnoic, apyretic, with the oxygen saturation within the normal range (97%). Over the course of hospitalization, the patient remained positive for SARS-CoV-2 infection. From day 53 to 146 he quarantined asymptomatic at home. During this period, viral RNA was clearly detectable in nasopharyngeal swabs collected at day 116, 125, and 146. Lymphoma evolution called for the need of autologous transplantation, so the patient was hospitalized for administration of therapy aimed to stem cell collection on day 146 (Cytorabine plus Rituximab) and day 158 (Rituximab). As shown in **Fig. 1**, SARS-CoV-2 has been continuously detected for as long as 222 days. During this period, SARS-CoV-2 RT-PCR showed a median RdRp gene Cycle threshold (Ct) of 25.07 (range from 17.8 to 33.3). Starting from day 237, he tested negative on seven consecutive sampling (last sampling was on day 270), indicating that SARS-CoV-2 infection was cleared.

To the best of our knowledge, this is the first described case where an immunocompromised symptomatic individual spontaneously recovers from COVID-19 in the absence of antiviral and/or convalescent plasma treatments.

### 3.2 Intra-host viral genetic diversity

Using deep sequencing data, we were able to follow the dynamics of the intra-host evolution during a SARS-CoV-2 prolonged infection (**Fig. S1**). Genetic screening of all samples revealed 610 intra-host single nucleotide variants (iSNVs) with allele frequency >10%, with nonsynonymous changes representing 408 (85%) out of 482 iSNVs detected in coding regions. The highest abundance of iSNVs were mainly scattered over ORF1ab and S genes. In particular, Orf1ab harbored the majority of the iSNVs detected (n=266) followed by the S (n=156), N (n=36), orf3a (n=21), E (n=2), and orf8 (n=1) gene, and noncoding regions (n=128). The comparison of iSNVs down to low frequencies among serial samples has revealed an elevated accumulation of SARS-CoV-2 intra-host variants emerged out of a population that existed previously. Patterns in the variant frequencies suggest a competition between virus populations carrying different mutations. For example, viruses with the spike deletion [S_Y144del (21991 del-TTA)] rose to high frequency at day 7, but were then quickly outcompeted by another population without this deletion from day 20. Many of the iSNVs with a small representation in the early samples appeared with almost the same frequencies also in later samples, suggesting that the competition between the intra-host populations was maintained. Notably, most iSNVs were remarkably stable while others showed a specific trend across sampling dates. For instance, eight iSNVs [5'UTR_236 (236 G->T), nsp3_D1639N (5180 G->A), Rdrp_L5230= (15952 C->T), methyltransferase_A6914V (21005 C->T), S_K182N (22108 A->C), S_L242del (22286 del-CTT), S_Q493K (23039 C->A), S_N501T (23064 A->C)] showed continuous increase in allele frequency becoming a stable change present in the consensus sequence of the last samples. In addition, these iSNVs with similar growth rates but appearances in different time points confirmed the co-existence of diverse viral populations within each sample.

Concerning nonsynonymous substitutions, genomic analysis of sequences revealed that since day 26 the virus showed non-conserved amino acid (aa) changes in a number of viral proteins. In particular, one aa mutation was observed in nsp3 (D1639N) and in methyltransferase (A6194V) (**Fig. S2A**). At the same time, we observed the occurrence of one deletion (L242del) and one mutation (Q493K) in the spike S1 region (**Fig. S2A and S2B**). These aa changes were represented in the consensus sequence of all consecutive samples until the infection was cleared. It is worth noting that the Q493K mutation has been rarely found elsewhere up to March 15$^{th}$ 2021, since only 30 out of 784883 (0.004%) genomes available on GISAID (https://www.gisaid.org/) showed the presence of K at the spike position 493. As shown in **Fig. S2B** and in **Fig. 1B**, a N501T mutation in the spike S1 region, firstly observed in the consensus sequence at day 48, initially appeared in 11.8% of the total reads at day 34 and then became predominant in all the subsequent sampling and being maintained till viral clearance. Interestingly, at day 125 we found that a N501Y sequence transiently dominated over the N501T (53.7% vs 45.1%, respectively) but disappeared soon after. In the last viremic sample collected at day 222, other three non-conserved aa substitutions were expressed in helicase (R5661C), exonuclease (V6207I), and orf3a (L108F). The sudden appearance of these nonsynonymous mutations in the master sequence at day 222 is likely to reflect virus evolution more than the emergence of previously concealed quasispecies.

Surprisingly, all dominant non-conserved aa substitutions or deletions we observed over time, even transiently, in the spike S1 region were already present in nasopharyngeal swabs collected as early as at day 0 and 7 (**Fig. 1B**). This finding highlights a fast changing also in the genetic characteristics of a key functional gene such as the S gene, further suggesting a dynamic replacement of genome subpopulations of related quasispecies within replicating ensembles.

**3.3 Phylogenetic analysis of consensus sequences**

In order to understand the evolution of SARS-CoV-2 and the dynamics of iSNVs in an immunocompromised host, we performed a phylogenetic analysis using viral consensus sequences obtained from 12 nasopharyngeal swabs collected between day 0 and day 222. The time stamped tree revealed the phylogenetic relationship among the 12 generated strains, showed that all of them belonged to an ancestral SARS-CoV-2 lineage (B.1.1) (**Fig. 2**). Our results suggest that sequences at day 0, 7, and 20 scattered on different branches of the phylogenetic tree. At the same time, sequences obtained from day 26 to 222 established an independent cluster. This finding further suggests an intra-host virus evolution, in which genomic diversity is relatively heterogeneous during the early stage of infection, but becoming more homogeneous over time as a consequence of virus adaptation to the immunocompromised host in search for a better infectivity or resistance to innate immune responses.

### 3.4 SARS-CoV-2 quasispecies competition *in vitro*

Replicating ensembles are subjected to episodes of competition. This is mostly due to the biosphere diversity in which viruses are installed and to their need of meeting with unique intracellular environments (Domingo et al., 2021). To test the hypothesis of a dynamic replacement of SARS-CoV-2 quasispecies due to competition mechanisms *in vivo*, we first isolated the dominant virus from nasopharyngeal swabs collected at day 0 ($MB61^{0}$) and at day 222 ($MB61^{222}$) and then tested its capability to replicate in two different human cancer cell lines as lung adenocarcinoma (Calu-3) and colon carcinoma (Caco-2). Quantitation of viral genomes in cell supernatants collected at 12, 24, 48, 72 and 96 h post infection (p.i.) by digital droplet PCR (ddPCR) did show a significant difference in the replication of the two isolates in Calu-3 cells at 12 h p.i., with a faster growing of $MB61^{222}$ than $MB61^{0}$ (**Fig. 3A**). At the same time, a significantly faster replication of $MB61^{222}$ was observed in Caco-2 cells at each time p.i., as compared to $MB61^{0}$ (**Fig. 3B**). Similar results were obtained by plaque assay quantification of viable virus (**Fig. S3A** and **B**). For coinfection experiments, Calu-3 and Caco-2 cells were infected with a mixture of each virus at a multiplicity of infection (MOI) of 0.01, and supernatant was collected at different time points for virus quantitation by next generation sequencing (NGS). To this aim, MB61$^{222}$ was distinguished from MB61$^{0}$ for its peculiar Q493K and N501T signatures in the S gene. Viral relative abundance of MB61$^{222}$ in comparison to MB61$^{0}$ was determined through the analysis of NGS reads. The percentage of reads carrying the nucleotide substitution characterizing MB61$^{222}$ was evaluated compared to the MB61$^{0}$ reads. As shown in **Fig. 3C**, quantification of RNA released over time shows that viral production is very similar in Calu-3 and Caco-2 cells. This data was also confirmed by plaque assay (**Fig. S3C**). However, as soon as 24 h after coinfection of Calu-3 cells, MB61$^{222}$ replication was significantly increased as compared to MB61$^{0}$. Approximately 10% of total virus detected in the supernatant of coinfected cells at the experimental endpoint (96 h) was ascribed to MB61$^{0}$, suggesting that MB61$^{222}$ actively replaced its competitor (**Fig. 3D**). Even more striking results were obtained in coinfection experiments performed in Caco-2 cells. In fact, a significantly higher replication activity of MB61$^{222}$ compared to MB61$^{0}$ was evident at as early as 12 h p.i., with MB61$^{222}$ representing approximately 70% of the released virus (**Fig. 3E**). Results obtained suggest that the capability of MB61$^{222}$ to replace MB61$^{0}$ in two different target cell lines might be ascribed to mechanisms of interference able to alter viral population dynamics in coinfected cells.

### 3.5 MB61$^{0}$ and MB61$^{222}$ isolates exploit different cell entry mechanisms

One possible difference in the ability of MB61$^{0}$ and MB61$^{222}$ to interact with target cells might be ascribed to the cell entry mechanisms. SARS-CoV-2 might enter target cells via endocytosis or PM fusion pathways in the presence of TMPRSS2, the proteolytic process of SARS-CoV-2 is completed at the plasma membrane, but in its absence the virus is endocytosed and sorted into endolysosomes, from which SARS-CoV-2 enters the cytosol via clathrin and cathepsin L. (Jackson et al., 2022). We investigated the entry mechanisms used by MB61$^{0}$ and MB61$^{222}$ in Calu-3 cells. We observed that TMPRSS2 inhibition obtained by Camostat was able to significantly reduce both MB61$^{0}$ and MB61$^{222}$ replication at 24 h p.i. (**Fig. 4A**). However, the antiviral effect of Camostat was transient, being the amount of released $MB61^0$ and $MB61^{222}$ at 48 h p.i. similar in cells treated or not with the drug.

Since SARS-CoV-2 might also use endocytic processes, we performed the inhibition of endocytosis via Chlorpromazine and Cathepsin L inhibitor III, that block clathrin-mediated endocytosis (Vercauteren, et al., 2010) and cathepsin L, respectively. As shown in **Fig. 4A**, inhibition of the clathrin-dependent early endosome formation significantly reduced $MB61^0$ replication. At the same time, cathepsin L significantly reduced $MB61^0$ replication. On the other hand, clathrin and cathepsin-L inhibitors did not exert any effect on $MB61^{222}$ replication. These results support a major involvement of the endocytosis entry mechanism for $MB61^0$, while $MB61^{222}$ mainly utilizes the PM fusion pathway.

In the effort to elucidate this different behavior of $MB61^0$ and $MB61^{222}$, we evaluated the expression of Interferon-inducible transmembrane proteins (IFITMs), that are intensely induced during viral infection and play a crucial role in virus restriction of endosomal entry (Winstone, et al., 2021). Simultaneously, we evaluated the level of LY6E expression, that is implicated in the control of the PM fusion pathway (Pfaender, et al., 2020). As shown in **Fig. 4B**, $MB61^{222}$ infection significantly enhanced the expression of IFITM1 and IFITM2, as compared to $MB61^0$ infection. At the same time, both isolates did not modify the expression of IFITM3 and LY6E. These results sustain a possible implication of IFITM1 and IFITM2 in the control of endosomal viral entry during SARS-CoV-2 infection. Indeed, $MB61^{222}$ mainly uses the PM fusion pathway and escapes this host cell defense mechanism. On the contrary, $MB61^0$ cannot escape this host antiviral response during $MB61^{222}$ co-infection, since it has endocytosis as main route of entry into the target cell.

### 3.6 Molecular modelling results

Mutation at position 501 (N501Y) rose along with the B.1.1.7 variant and is associated primarily with an increase in hACE2 affinity and increased infectivity (Bayarri-Olmos et al., 2021; Liu et al., 2021; Ramanathan et al., 2021; Tian et al., 2021). The role of mutation at position 493 has been mostly linked to immune evasion (Thomson et al., 2021). Molecular modelling was then applied to gain better inside the role of Q493K and N501T mutations in providing a better interaction with hACE2 and provide a rationale to explain the slightly faster replicative activity of MB61$^{222}$ as compared to MB61$^{0}$.

Crystal structures of SARS-CoV-2 RBD-hACE2 complex show that the contact regions (CR) of the RBD are mainly concentrated in the Receptor Binding Motif (RBM) denoted as CR1, CR2 e CR3. Q493K and N501T mutations are located in CR2 and CR3 respectively. Protein-protein association between hACE2 receptor and RBD consists of a hydrogen-bonding network and hydrophobic interactions. Molecular dynamics of binary complexes of the RBD with hACE2 were carried out to investigate whether mutations can influence the interfacial interactions. Root mean square deviation (RMSD) was assessed during the 200-ns of MD simulation runs for RBD and RBM of SARS-CoV-2 (wild-type; WT) and SARS-CoV-2 (Q493K + N501T) (mutant; Mut) systems. As shown in **Fig. S4**, there were no significant differences between SARS-CoV-2 WT and Mut in the RBD, RBM, hACE2 and the contact regions (H1, H2, H18 and b-turn) of hACE2 during the simulations, thus indicating stable MD simulations for both spikes in these regions. Also, the root mean square fluctuation (RMSF) values of hACE2 and the RBD domain of CA atoms in both SARS-CoV-2 WT and Mut complexes were comparable and showed limited fluctuations.

Comparison at the key interfacial interactions between hACE2 and the RBD of SARS-CoV-2 shows that SARS-CoV-2 WT (**Fig. 5A**) and SARS-CoV-2 Mut (**Fig. 5B**) have diverse epitope features. In the SARS-CoV-2 Mut RBD-hACE2 the replacement of Q493K maintains the hydrogen bond between 493 residue of CR2 and E35 of hACE2 as in the WT, but the greater flexibility of the lysine side chain compared to glutamine generates a new hydrogen bond between 493K and D38 of hACE2. The new hydrogen bond removes D38 from the delicate hydrogen bond network of CR3 and induces a rearrangement of the interactions in this contact region. The replacement of N501T generates a loss of six hydrogen bonds in CR3, but a new hydrophobic patch is generated among

T500, V503 of CR3 and Q325, N330 of hACE2 and these new interactions extend the terminal contact region of CR3 by engaging the helix 18 (H18) close to the beta strands of hACE2 (**Fig. 5B**). The hydrogen networks observed in the representative conformations obtained from the molecular dynamics are in agreement with the frequency of the hydrogen bonds calculated during the simulation (**Fig. S5**). Furthermore, Q493K substitution inserts a positive charge and the lysine forms two new saline bridges among K493 of RBD and E35 and D38 of hACE2. A comparison of the surface electrostatic potential identifies a positive charged patch on the SARS-CoV-2 Mut RBD-hACE2 that is absent on the SARS-CoV-2 WT RBD-hACE2 (**Fig. 5C** and **Fig. 5D**). K493 contributes to form positive electrostatic surface in the SARS-CoV-2 Mut (**Fig. 5D**). The present study reveals a reorganization of key interfacial interactions between hACE2 and the SARS-CoV-2 Mut RBD. In addition, Q493K conversion in the SARS-CoV-2 Mut RBD creates a positive electrostatic patch that results in greater electrostatic complementarity than that of the SARS-CoV-2 complex.

### 3.7 MB61$^{0}$ and MB61$^{222}$ induce peculiar RNA sensors pathway activation

A differential pathway for viral entry might induce substantial differences in viral RNA sensing by the infected cells since the different timing and localization for genome released engages different intracellular RNA sensors.

We investigated both cytosolic (RIG-I and MDA5) and endosomal (TLR3, TLR7, and TLR8) RNA sensors to identify possible differences between MB61$^{0}$ and MB61$^{222}$ SARS-CoV-2 isolates. As shown in **Fig. 6**, the cytosolic RIG-I RNA sensor was significantly up-modulated as mRNA (**Fig. 6A**) and protein (**Fig. 6B**) expression by MB61$^{222}$ only. The endosomal TLR3 and TLR7 RNA sensors were triggered at the mRNA (**Fig. 6A**) and protein (**Fig. 6B**) level by both MB61$^{0}$ and MB61$^{222}$. Interestingly, MB61$^{222}$ induced a higher up-modulation of TLR7 than MB61$^{0}$. On the contrary, MDA5 and TLR8 were not induced by both SARS-CoV-2 isolates. These data suggest that MB61$^{222}$ infection induces the transcription and transduction of the cytosolic RIG-I RNA

sensor and of TLR3 and TLR7 endosomal RNA sensors. On the contrary, MB61$^{0}$ infection enhances TLR3 and TLR7 endosomal RNA sensors only, the latter at a lower extent as compared to MB61$^{222}$.

The ability of MB61$^{222}$ to engage cytosolic RIG-I RNA sensor suggests a differential down-stream transduction as compared to MB61$^{0}$ that enhances endosomal TLR3 and TLR7 RNA sensors only. Among the transcriptional factors involved in RNA sensing by viral infected cells, IRF3 and NF-κB play a central role (Schmitz et al., 2014). In particular, IRF3 protein is involved in the production of interferons (Yanai et al., 2018), whereas NF-κB is mainly employed in the induction of the proinflammatory response (Liu et al., 2017). Even if both IRF3 and NF-κB are reported to be crucial in RNA sensing signaling, they are differentially induced by different RNA sensors. In fact, while endosomal RNA sensors activation leads mainly to NF-κB recruitment, the cytosolic RNA sensor RIG-1 typically activates both NF-κB and IRF3 signal (Nguyen et al, 2020). As shown in **Fig. 6C**, a significant activation of IRF3 and NF-kB was observed in MB61$^{222}$-infected cells. On the other hand, MB61$^{0}$-infected cells showed an enhanced NF-kB transcription only. These data agree with the differential RNA sensors induction by the two SARS-CoV-2 isolates. MB61$^{222}$ was found to significantly enhance RIG-I, TLR3 and TLR7 RNA sensors and consequently both IRF3 and NF-kB transcription and phosphorylation (**Fig. 6D**). On the contrary, MB61$^{0}$ induces TLR3 and TLR7 RNA sensors and the down-stream factor NF-kB transcription and phosphorylation (**Fig. 6D**).

### 3.8 MB61$^{0}$ and MB61$^{222}$ SARS-CoV-2 infection triggers different mediators of the innate immune response

It is known that depending on the ability of the cell to sense the presence of an infection, different soluble factors, such cytokines and interferons (IFNs), are released. NF-κB and IRF3 are essential to mediate antiviral actions through the expression of virus-induced and IFN-stimulated genes. We analyzed the expression levels of cytokines, type-I (IFN-α and IFN-β) and type II (INF-γ) IFNs in Calu-3 cells infected with $MB61^0$ or $MB61^{222}$.

As reported in **Fig. 6E**, $MB61^{222}$ infection significantly increased the mRNA transcription of the pro-inflammatory cytokines IL-1β and IL-6 in comparison with uninfected and $MB61^0$-infected Calu-3. Similarly, IL-1β and IL-6, as well as TNF-α secretion were significantly increased in $MB61^{222}$ – but not in $MB61^0$-infected cells (**Fig. 6F**). IFN-α, IFN-β, and IFN-γ were significantly induced during $MB61^{222}$ infection as mRNA transcription (**Fig. 6G**) and protein secretion (**Fig. 6H**) as compared to $MB61^0$-infected cells. On the contrary, $MB61^0$ infection was not found to affect IFN-α and IFN-β transcription and secretion (**Fig. 6G** and **Fig. 6H**). However, $MB61^0$ infection was found to significantly induce IFN-γ secretion as compared to uninfected cells (**Fig. 6H**). These results are in agreement with the differential effects that the two isolates promote on RNA sensors.

Altogether, our results show that $MB61^{222}$ enters into the target cell via both PM fusion and endocytosis, enhancing cytosolic (RIG-I) and endosomal (TLR3, TLR7) RNA sensors. The consequent phosphorylation of both IRF3 and NF-kB transcription factors induces the secretion of proinflammatory cytokines (IL-1β, IL-6 and TNF-α), and IFNs (IFN-α, -β, and -γ), under RIG-I and TLRs control. On the contrary, $MB61^0$ enters into the target cell via endocytosis, inducing TLR7 and TLR3 RNA sensors, with the consequent activation of NF-kB and IFN-γ secretion.

**3.9 Proteome analysis of SARS-CoV-2-infected Calu-3 cells**

Proteomics profiles of Calu-3 cells infected or not with $MB61^0$ or $MB61^{222}$, collected at 12, 24 and 48 h p.i. were analyzed. Through a shotgun label-free platform, based on the coupling of nanoliquid chromatography and high-resolution mass spectrometry (nLC-hrMS/MS), 42 proteomics runs were acquired from the 7 examined conditions. A total of 5113 distinct proteins were identified (**Table S1**) with at least one unique peptide, molecular weights ranging from 2 to 3990 kDa and isoelectric points from 3.8 to 12.3. The alignment of all protein lists obtained was carried out on the basis of identified proteins and related peptide spectrum match values (PSMs), that represent the number of mass spectra attributed to them and indirectly their abundance in the samples. Then, for each experimental condition three lists were created normalizing and averaging the peptide spectrum match values (aPSMs) of the proteins identified with high confidence with at least one unique peptide using a total signal normalization method. The linear discriminant analysis (LDA) was applied using the proteomics lists and 431 statistically significant proteins presenting a p value ≤ 0.0001, were extracted as differentially expressed proteins (DEPs) (**Table S2**). As shown in **Fig. 7A**, a clear separation and a different behavior between Calu-3 and $MB61^{0}$ or $MB61^{222}$-infected cells, which generate two main branches, was appreciable by the distribution of heatmap colors. A further subgrouping is also evident according to the time p.i. and to the isolate considered. Of note, Calu-3 cells infected with $MB61^{222}$ and collected at 48 h p.i. are strikingly different from all the other ones examined conditions.

Selected DEPs were combined with the *Homo sapiens* protein-protein interaction network to highlight molecular functions, biological processes and pathways affected by virus infection, at different time. Following this strategy, a network model of 421 proteins connected by 4509 interactions was built. Globally, these proteins were grouped across in 51 functional modules (**Fig. S6**). The evaluation of the protein pathways showed a differential induction by the two isolates, supporting our results. In particular, as shown in **Fig. 7B**, $MB61^{222}$ induced RNA sensing proteins (IFIT1, IFIT2 and IFI16) by $MB61^{222}$ (Vladimer et al., 2014) as well as IFN-induced genes (IFI44L, RSAD2, OAS2, OASL, MX1 and SATS1). The ability of $MB61^{222}$ to counteract this host antiviral response might be, at least in part, sustained by the induction of: i) CD59 (Wei et al., 2017), a negative regulator of the terminal pathway of the complement system by binding to the C8/C9 factors; ii) granulin, that binds directly to tumor necrosis factor receptors (TNFRs) and disturbed the TNFα-TNFR interaction (Tang et al., 2011); iii) SLFN5, which represses IFN-induced STAT1 transcriptional activity (Arslan et al., 2017); and iv) nucleobindin 1 (NUCB1), that has been shown to inhibit ATF6 activity (Madden et al., 2019). On the contrary, $MB61^{0}$ was not found to promote synthesis of proteins with antiviral activity.

The enhanced endocytic activity during MB61$^{0}$ infection is supported by the up-modulation of: HIP1R (Chen and Brodsky, 2005) and CLINT1, that interact with clathrin; GATD1 and IGF2R, implicated in intracellular trafficking; and by the focal adhesion proteins (MUCSAC, TLN1, LAMC2, LAMB3, FHL2, GLG1), that might facilitate SARS-CoV-2 adhesion and integrin signaling, which terminates with endosomal trafficking. To facilitate particle uncoating, MB61$^{0}$ induced MAPRE2, NUMA1 and MACF1 that sustain the microtubule-associated particle uncoating. The decreased PM fusion pathway during MB61$^{0}$ infection might by also determined by the increased expression of SERPINB1 by infected cells, which is predicted to reduce the ability of TMPRSS2 to facilitate SARS-CoV-2 entry into airway epithelial cells (Stanton et al., 2020). Interestingly, MB61$^{0}$ also enhanced the expression of proteins (GDI1, SEC22B, COPG1, HOOK1, ARFIP1, EMC1, RRBP1, SRP68) involved in the formation of endoplasmic reticulum (ER)-derived perinuclear interconnected membrane structures that contain double-membrane vesicles (DMVs), in which SARS-CoV-2 RNA synthesis occurs (Snijder et al. 2020). The use of endocytosis also by MB61$^{222}$ is supported by the expression of ARPC1B, TES and GC, that facilitate cargo enrichment and budding from the endosomal membrane to generate recycling vesicles. It is worth noting that MB61$^{222}$ may require this process for receptor recycling (Al-Saleem et al., 2019). Alternatively, the increased expression of DYNLL2, that controls retrogade vescicle transport, may facilitate virus trafficking to replication sites, or may serve to deliver cargoes to replication sites. The formation of DMVs during MB61$^{222}$ infection is also supported by the induction of REEP5, that interacting with SARS-CoV-2 structural M protein, plays a crucial role in viral morphogenesis.

## 4. Discussion

Quasispecies provide an advantage to SARS-CoV-2 for achieving the best fitness during prolonged intra-host evolution by changing the genetic characteristics of key functional genes. Our data show the existence of an ensemble of minor mutants in the early biological samples obtained from an immunocompromised patient and their dynamic interplay with the master mutant during a persistent and productive long-term infection. After 222 days of active viral replication *in vivo*, we highlighted the replacement of the original master mutant by a minor mutant, namely $MB61^{222}$, expressing two critical mutations in spike, namely Q493K and N501T. This mutated spike shows a greater affinity to ACE2 than the original spike, which may explain the acquired capability of $MB61^{222}$ to gain access into target cells mainly by the PM fusion pathway than endocytosis, which characterizes the entry mechanism used by the previous master mutant named $MB61^{0}$. In this shifting in the entry route preference, we cannot exclude the role played by other spike mutations acquired by $MB61^{222}$ during time. The enhanced endocytic activity during $MB61^{0}$ infection is supported by the up-modulation of HIP1R (Chen and Brodsky, 2005) and CLINT1, that interact with clathrin, GATD1 and IGF2R, implicated in intracellular trafficking and by the focal adhesion proteins (MUCSAC, TLN1, LAMC2, LAMB3, FHL2, GLG1), that might facilitate $MB61^{0}$ adhesion and integrin signaling, which terminates with endosomal trafficking. On the contrary, the decreased PM fusion pathway during $MB61^{0}$ infection might by determined by the increased expression of SERPINB1 by infected cells, which is predicted to reduce the ability of TMPRSS2 to facilitate SARS-CoV-2 entry into airway epithelial cells (Stanton et al., 2020). These findings, in agreement with previous observations (Nelson et al., 2021; Starr et al., 2020), may support the significant increase in infectivity and replication of $MB61^{222}$ as compared to $MB61^{0}$ we observed at early time (12-24 h) p.i.. It is worth noting that the expression of ARPC1B, TES and GC, that facilitate cargo enrichment and budding from the endosomal membrane to generate recycling vesicles, might facilitate ACE2 receptor recycling and further contribute to enhance $MB61^{222}$ infectivity (Al-Saleem et al., 2019).

Different modality of SARS-CoV-2 entry are known to induce activation of different RNA sensors (Bortolotti et al., 2021; Mdkhana et al, 2021). The ability of MB61$^{222}$ to engage cytosolic RIG-I RNA sensor leads to a differential down-stream transduction as compared to MB61$^{0}$, that enhances endosomal TLR3 and TLR7 RNA sensors only. Consequently, IFN-α, IFN-β, and different pro-inflammatory proteins are significantly induced during MB61$^{222}$ infection. On the contrary, MB61$^{0}$ infection did not significantly promote any activation of the innate immune system but INF-γ transcription and secretion only.

In a recent study, (Thorne et al. 2021) Thorne et al. revealed that mutations outside S gene, including non-coding changes, are important in SARS-CoV-2 adaptation through enhanced innate immune escape. In particular, they discovered an upregulation of N, N* and Orf9b expression in Alpha (B.1.1.7) variant. These proteins, encoded by N alternative reading frame, are key viral innate antagonists. They suppose that the increase in Orf9b transcription derives from one-base deletion at 28,271 found in Alpha and Delta (B.1.617.2) variants and A>T substitution in the Omicron (B.1.1.529) variant, which weakens their Kozak translation initiation complex enhancing leaky scanning of the N gene. Additionally, an increase in Orf9b protein expression is likely influenced by 3-nucleotide mutation at 28,280-28,282 GAT>CTA, which modifies the Alpha Orf9b Kozak context introducing an enhanced transcriptional regulatory sequence (TRS). We evaluated MB61$^{0}$ and MB61$^{222}$ N gene sequences but, as shown in **Fig. S7**, we did not detect any of the above described nucleotide changes, thus excluding their role in establishing different innate immune responses evoked by the two isolates.

It has been previously suggested (Parker et al. 2021) that the 3-nucleotide mutation at 28,881-28,883 GGG>AAC found in Alpha, Gamma and Omicron variants creates a novel TRS for N* transcription. Increased N* protein levels are supposed to act as innate immune antagonists by sequestering dsRNA. However, this mechanism cannot explain the different capability of our isolates to trigger innate immunity since both MB61$^{0}$ and MB61$^{222}$ show these nucleotide changes in their N gene sequence (**Fig. S7**).

Interestingly, co-infection of two human cell lines of different origin with our two SARS-CoV-2 isolates highlighted the dramatic predominance of MB61$^{222}$ replication over that of MB61$^{0}$. This may be attributed to a faster replication activity of MB61$^{222}$ compared to MB61$^{0}$ and to the capability of MB61$^{222}$ to activate RNA sensors and trigger potent innate immune responses. IFN-stimulated gene products are involved in SARS-CoV-2 entry steps, as IFITMs (Winstone et al., 2021; Shi et al., 2021). Four members of these proteins (IFITM1, IFITM2, IFITM3 and IFITM5) are strongly induced by type I and type II IFNs and known to act as antiviral molecules against Influenza A viruses, Flaviviruses, Filoviruses and SARS-CoV (Szabo and Bugge, 2008; Szabo and Bugge, 2011). More recently, IFITM2 was found to restrict SARS-CoV-2 entry occurring by endocytosis (Winstone et al, 2021). Although the mechanisms are still unknown, IFITM2 was found to prevent SARS-CoV-2 to traverse the endosomal membrane to access cellular cytoplasm. Interestingly, this did not occur if SARS-CoV-2 gains access into the target cells by PM fusion at the plasma membrane (Huang et al, 2011). We observed an increased expression of IFITM1 and IFITM2 during MB61$^{222}$ infection, suggesting a possible implication of IFITMs in selectively impairing MB61$^{0}$ replication. Indeed, the ability of MB61$^{222}$ to shift viral entry towards the PM fusion pathway may allow it to escape the host cell blocking of endosomal viral entry. On the contrary, MB61$^{0}$ is a target for the antiviral activity of IFITM1 and IFITM2 because it lacks the possibility to use the PM fusion pathway. These findings suggest that during coinfection of a cell with the two SARS-CoV-2 isolates, MB61$^{222}$ may dominate by triggering an IFN-mediated activity aimed to neutralize MB61$^{0}$ entry and replication.

MB61$^{222}$ is able to evoke a potent innate immune response, which consists in the production and release of type I and II IFNs and different pro-inflammatory cytokines. Usually, early host innate responses protect airway cells from infection and limit viral dissemination (Sposito et al., 2021). The capability of MB61$^{222}$ to actively replicate in such an unfavorable microenvironment supports the hypothesis that it can escape host innate responses by activating still unknown antagonists. Indeed, viral innate resistance is likely to have contributed to prolonged *in vivo* viral shedding in the immunocompromised host, whose antiviral defenses were essentially based on innate immunity. This hypothesis is also consistent with reports of enhanced innate evasion and prolonged shedding of the Alpha variant (Calistri et al., 2021; Kissler et al., 2021; Thorne et al., 2021). It is worth noting that the inflammatory switch operated by $MB61^{222}$, may have been evolved to also enhance viral spreading at the site of infection as already evidenced for the Alpha variant (Davies et al., 2021; Scientific Advisory Group for Emergencies, 2021). Altogether, our findings highlight the importance of innate immune evasion in the still ongoing process of SARS-CoV-2 evolution aimed to find the optimal adaptation to replicate and disseminate into the human host. However, the role of other mutations than N501T and Q493K acquired by $MB61^{222}$, in increasing viral replication, promoting innate immune evasion and changing the cytokine profile, cannot be completely ruled out.

In conclusion, this study further points to uncontrolled and prolonged infection in immunocompromised patients as the condition that may have shaped the emergence of SARS-CoV-2 variants with a higher capability than their ancestors to rapidly spread around the world. Interestingly, our data show a competition among virus quasispecies to hold a dominant replicative activity and spreading by manipulating the host cell innate immunity. This finding subverts the idea of quasispecies as an advantage mutation pool for the epidemic variants to find the best genetic fitness during intra-host evolution and introduces a new concept of quasispecies fighting for host dominance by taking benefit from the cell machinery to restrict the productive infection of the other competitors in the viral ensemble. This may explain, at least in part, the extraordinary rapid worldwide turnover of a VOC over the original pandemic strain observed in the COVID-19 pandemia. The preexistence of potential fitness mutants calls for the importance of studying the mutation spectra of different hosts, and minor mutants in particular, to predict the emergence of new variants with neutralizing antibody or antiviral drug escape ability.

## Data availability

Genomic data reported in this study are available at Global Initiative on Sharing All Influenza Data (GISAID).

Accession numbers:

EPI_ISL_2484209 (hCoV-19/Italy/LOM-UNIBS-MB61-Day0/2020)
EPI_ISL_2484210 (hCoV-19/Italy/LOM-UNIBS-MB61-Day7/2020)
EPI_ISL_2484211 (hCoV-19/Italy/LOM-UNIBS-MB61-Day20/2020)
EPI_ISL_2484212 (hCoV-19/Italy/LOM-UNIBS-MB61-Day26/2020)
EPI_ISL_2484213 (hCoV-19/Italy/LOM-UNIBS-MB61-Day34/2020)
EPI_ISL_2484214 (hCoV-19/Italy/LOM-UNIBS-MB61-Day41/2020)
EPI_ISL_2484215 (hCoV-19/Italy/LOM-UNIBS-MB61-Day48/2020)
EPI_ISL_2484216 (hCoV-19/Italy/LOM-UNIBS-MB61-Day116/2020)
EPI_ISL_2476401 (hCoV-19/Italy/LOM-UNIBS-MB61-Day125/2020)
EPI_ISL_2484217 (hCoV-19/Italy/LOM-UNIBS-MB61-Day146/2020
EPI_ISL_2484218 (hCoV-19/Italy/LOM-UNIBS-MB61-Day158/2020)
EPI_ISL_2484219 (hCoV-19/Italy/LOM-UNIBS-MB61-Day222/2020)

## Funding

This research was supported (to PM) by Infrastructure CNR.Biomics PIR01_00017 project, funded by the MIUR_PON R&I 2014-2020_Elixir.

## Conflict of interest

The authors declare no conflict of interest.

## Figure legends

**Figure 1. Timeline of SARS-CoV-2 infection and frequencies of longitudinal variants across the SARS-CoV-2 genome in viral quasispecies population**

(A) The line graph indicates the SARS-CoV-2 RT-PCR Ct values in nasopharyngeal swabs. Blue in boxes highlights the time of sample collection. Green in box indicates the time of transplantation. Shaded areas are period of hospitalization (light blue corresponds to the COVID-19 phase). (B) Table showing the alternative allele frequencies of intra-host single nucleotide variants (iSNVs) and all amino acid substitutions. To minimize false discoveries, an iSNV was identified at genome positions with >4000-fold sequencing coverage and with at least four reads supporting the nucleotide substitution. Amino acid substitutions at the identified iSNV sites were measured as the proportion of paired-end mapped reads with the alternative amino acid. Variants types were highlighted in different colors scale. Mutations in gray were in the original day 0 sample and persisted.

**A**

**B**

| Mutation | Day 0 | Day 7 | Day 20 | Day 26 | Day 34 | Day 41 | Day 48 | Day 116 | Day 125 | Day 146 | Day 158 | Day 222 |
|---|---|---|---|---|---|---|---|---|---|---|---|---|
| 5'UTR_37 | 99,7% | 99,8% | 99,8% | 99,7% | 99,9% | 99,9% | 99,8% | 99,8% | 99,5% | 99,7% | 99,9% | 99,7% |
| 5'UTR_236 | 1,4% | 1% | 0,8% | 1,2% | 1% | 1,5% | 1,1% | 91,6% | 42,1% | 83,3% | 92,5% | 99,5% |
| 5'UTR_241 | 99,6% | 99,6% | 99,9% | 99,4% | 99,6% | 99,4% | 99,2% | 98,9% | 98,9% | 99,6% | 100% | 99% |
| nsp3_D1639N | 0% | 11,3% | 68,8% | 93,8% | 100% | 100% | 100% | 100% | 45,3% | 70% | 100% | 100% |
| Rdrp_P4715L | 99,5% | 99,8% | 99,5% | 99,6% | 99,2% | 99,6% | 99,7% | 99,6% | 99,5% | 99,7% | 99,7% | 99,5% |
| helicase_R5661C | 0% | 0% | 0,1% | 0,1% | 0,1% | 0% | 0,1% | 0,1% | 4% | 0,1% | 0,1% | 99,7% |
| exonuclease_V6207I | 0% | 0,1% | 0,1% | 0,0% | 0,1% | 0% | 0,1% | 0,1% | 3,5% | 0% | 0,2% | 99,4% |
| methyltransferase_A6914V | 0% | 7,3% | 62,0% | 93,6% | 97,8% | 99,2% | 97,6% | 99,1% | 99,5% | 89,8% | 99,4% | 99,7% |
| S_Y144del | 2,4% | 77,8% | 10,9% | 6,1% | 2,8% | 0,9% | 1% | 3,3% | 0,5% | 13,1% | 0,1% | 1,3% |
| S_K182N | 0,1% | 0,1% | 0,1% | 0,1% | 0,6% | 0,1% | 0,2% | 63,1% | 42% | 83% | 99,6% | 99,3% |
| S_L242del | 0,5% | 10,7% | 68,9% | 94,5% | 97,8% | 99,3% | 98,8% | 99,0% | 99,7% | 79,3% | 99,1% | 99,5% |
| S_Q493K | 0,1% | 7,7% | 61,8% | 93,4% | 97,5% | 99,7% | 98,9% | 99,6% | 98,5% | 73% | 99,6% | 99,6% |
| S_N501Y | 0,3% | 0,3% | 0,2% | 0,3% | 0,4% | 8,9% | 0,3% | 8,7% | 53,7% | 0,8% | 0,6% | 0,1% |
| S_N501T | 0,1% | 0,8% | 1% | 2% | 11,8% | 7% | 86% | 91,2% | 45,1% | 73,2% | 99,3% | 99,8% |
| S_D614G | 99,7% | 99,7% | 99,7% | 99,6% | 99,6% | 99,8% | 99,7% | 99,7% | 99,8% | 99,8% | 99,5% | 99,7% |
| ORF3a_L108F | 0% | 0,1% | 0% | 0,1% | 0,1% | 0,9% | 0% | 0% | 0,1% | 0% | 0% | 98,8% |
| N_R203K, G204R | 99% | 98,9% | 98,7% | 98,8% | 98,4% | 98,9% | 98,9% | 98,5% | 98,6% | 98,8% | 98,4% | 98,6% |

ACCEP

**Figure 2. Phylogenetic tree of the B.1.1 isolated sequences**

Time-resolved maximum likelihood tree including the 12 genomes obtained from the immunocompromised patient plus 2419 sequences representative of the SARS-CoV-2 lineage B.1.1 globally circulating until November 2021. Sequences belonging to the immunocompromised patient are identified by red circles. Highlighted is the clade containing the 9 clustering sequences isolated from day 26 to 222, and their most proximal ancestor sequence (black circle).

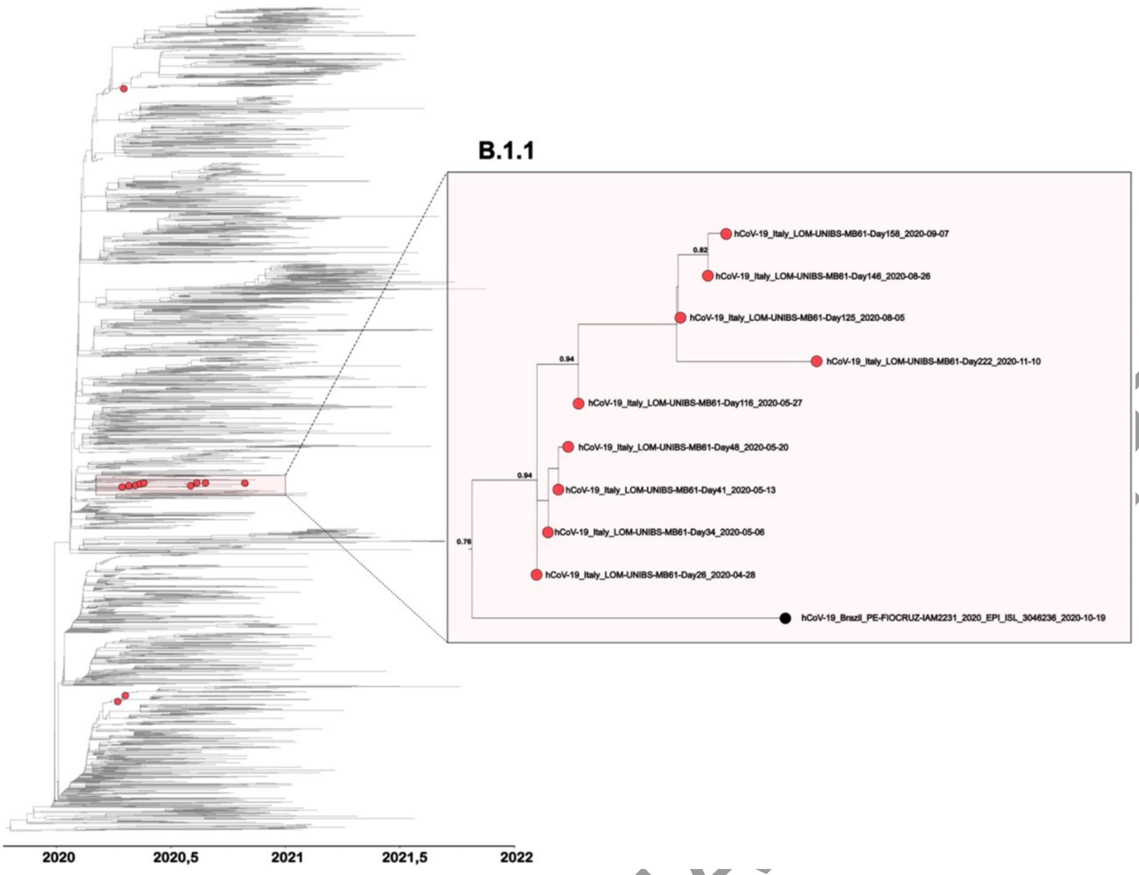

**Figure 3. Growth kinetics of the MB61$^{0}$ and MB61$^{222}$ patient's isolates in Calu-3 and Caco-2 cell lines.** (A) Human lung epithelial cell line Calu-3 and (B) Human Colorectal Adenocarcinoma cell line Caco-2 cells were infected with MB61$^{0}$ or MB61$^{222}$ at a MOI of 0.01. Supernatants from infected cells were harvested at designated time points for assessment of virus titer by ddPCR. Data shown are the mean and the standard error of the mean for three independent replicates. Student's unpaired two-tailed t-test was used to determine statistical significance for each time point (*$p < 0.05$, **$p < 0.01$, **** $p < 0.0001$). (C-E) *In vitro* simultaneous coinfection of Calu-3 and Caco-2 cell lines with MB61$^{0}$ and MB61$^{222}$. Calu-3 and Caco-2 cell lines were inoculated simultaneously with the MB61$^{0}$ and MB61$^{222}$ patient's isolates at a MOI of 0.01 each. (C) Supernatants from infected cells were harvested at designated time points for assessment of virus titer by ddPCR. Data shown are the mean and the standard error of the mean for three independent replicates. The statistical analysis was performed using a two-way ANOVA and Bonferroni's post-test was used to compare data. No significant difference in virus titer was observed between the two cell lines at any of the checked time points. Supernatants from infected Calu-3 (D) or Caco-2 (E) cells were collected at designated time points and then subjected to whole genome sequencing. Graphs show the percentage of $MB61^{222}$ versus percentage of $MB61^{0}$ at each of the time points (% viral fraction). The % of viral fraction was defined as the number of reads endowing the specific signatures of the $MB61^{222}$ patient's isolate. Data shown are the mean and the standard deviation for two replicates. The statistical analysis was performed using a two-way ANOVA and Bonferroni's post-test was used to compare data (**** $p < 0.0001$).

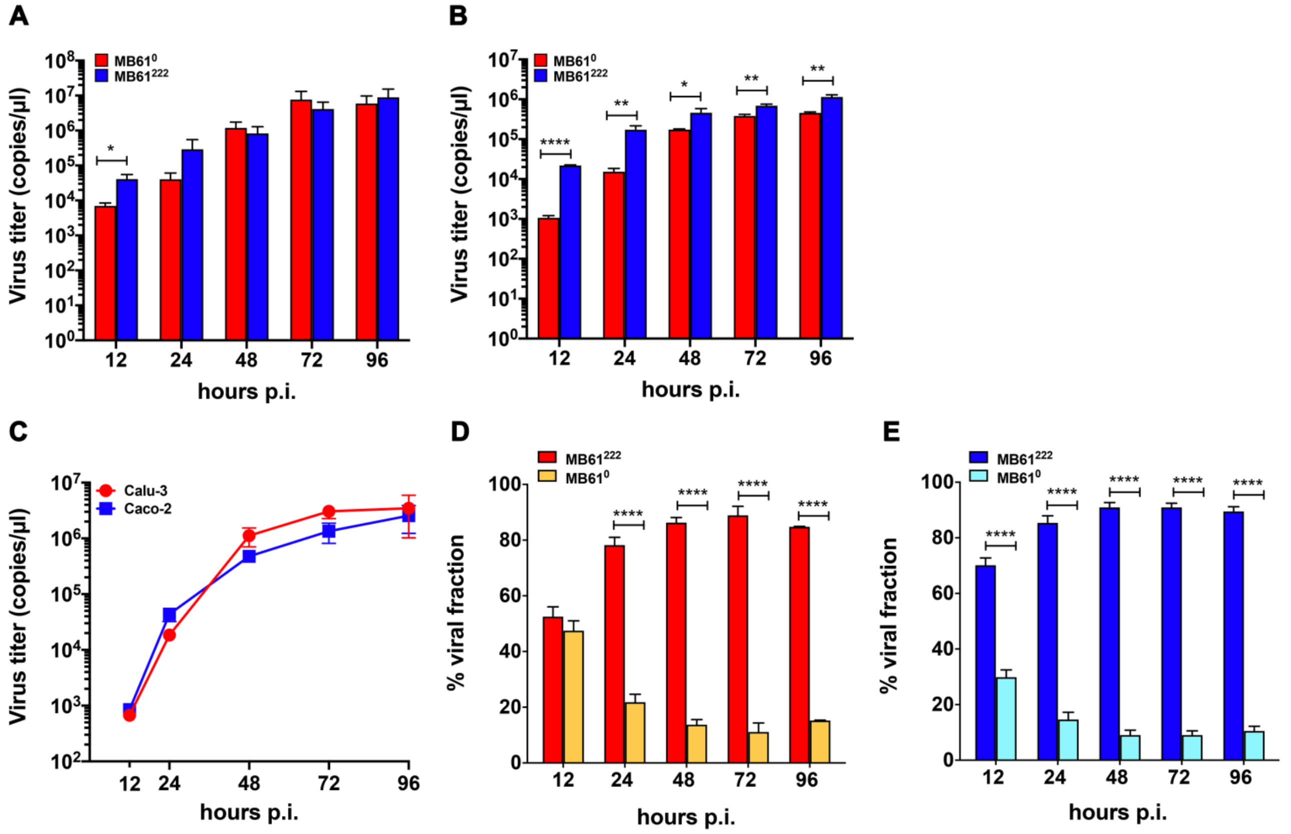

**Figure 4. $MB61^{0}$ and $MB61^{222}$ use different pathways to entry into Calu-3 cells.** (A) Calu-3 cells were infected with $MB61^{0}$ or $MB61^{222}$ at MOI of 0.01 and treated with TMPRSS2, clathrin, or cathepsin L inhibitors. Data shown are the mean and the standard deviation of the mean for three independent experiments. Student's unpaired two-tailed t-test was used to determine statistical significance between the two isolates for each inhibitor (**** $p < 0.0001$). (B) Gene expression of selected SARS-CoV-2 entry factors IFITM1, 2 and 3 and LY6E in Calu-3 cells infected with $MB61^{0}$ or $MB61^{222}$. Gene expression was determined by quantitative Real-Time PCR and normalized to not infected Calu-3 cells. Data shown are the mean and the standard deviation of the mean for three independent experiments. Student's unpaired two-tailed t-test was used to determine statistical significance between the two isolates for each inhibitor (* $p < 0.05$; ** $p < 0.01$).

**Figure 5. Spike RBM contact regions with hACE2 and surface electrostatic potential introduced by the Q493K and N501T mutation.**

RBM-hACE2 complex and the three contacts regions at the N-terminal end of hACE2 (CR1), the central region (CR2) of RBM, and the β-turn contact region of hACE2 (CR3) are shown. The key interfacial interactions between hACE2 and the RBM of SARS-CoV-2 WT (A) and SARS-CoV-2 Mut RBD (B) in the three contact regions are shown. Key hydrogen bonds are highlighted with dashed lines and hydrophobic contacts are shaded in yellow. Electrostatic interactions of SARS-CoV-2 WT RBD-hACE2 (C) and SARS-CoV-2 Mut RBD-hACE2 (D). Salt bridge is shown in yellow dot line, hydrophobic interactions are shaded in yellow sticks and electrostatic potential maps are colored in blue to indicate positive potential and red for negative potential.

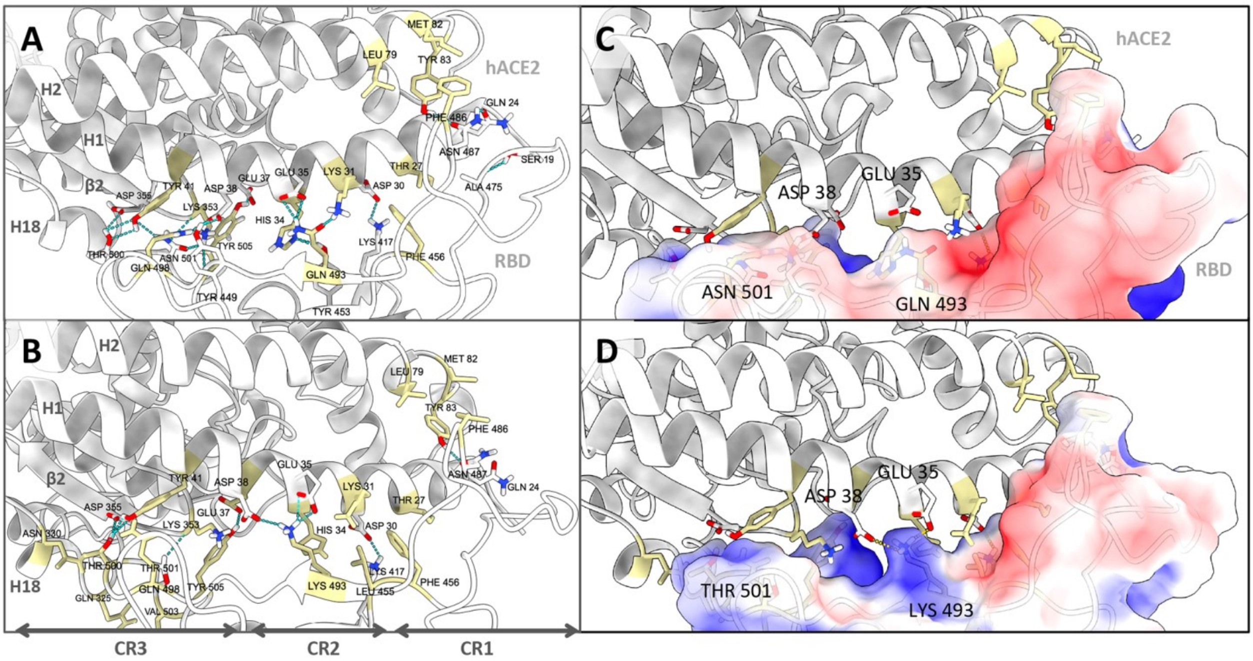

**Figure 6. RNA sensors in Calu-3 cells following MB61$^{0}$ or MB61$^{222}$ infection.** (A) Calu-3 cells were infected with MB61$^{0}$ or MB61$^{222}$ at a MOI of 0.01 for 1 h at 37 °C. Thereafter, the cells were washed and cultured for 48 h. Levels of expression of RIG-1, MAD5, TLR3, TLR7, and TLR8 were quantified as fold increase in comparison with not infected cells. (B, left panel) Western blot analysis of RIG-I, TLR3, TLR7 and β-actin in Calu-3 infected with MB61$^{0}$ or MB61$^{222}$ at a MOI of

0.01 The molecular weights were determined by protein ladder. RIG-I, TLR3 and TLR7 were evidenced at 100-116 kDa and β-actin at 44 kDa. (B, right panel) Evaluation of protein expression by densitometry, normalized on β-actin content. (C) Levels of NF-κB and IRF3 expression in $MB61^{0}$ or $MB61^{222}$-infected Calu-3 cells in comparison with not infected cells. (D) Levels of total and phosphorylated IRF3 and NF-κB in Calu-3 cells infected with $MB61^{0}$ or $MB61^{222}$ isolates and analyzed 48 h p.i.. Levels of (E) mRNA transcription and (F) protein expression of cytokines (IL-1α, IL-1β, IL-4, IL-6, TNF-α) are reported at 48 h p.i.. The amount of cytokines at 24 h p.i. were under the detection limit of the assays. Levels of (G) mRNA transcription and (H) protein expression of IFN-α, IFN-β, and IFN-γ are reported at 48 h p.i.. The amount of IFNs at 24 h p.i. were under the detection limit of the assays. Data shown are the mean and the standard deviation of the mean for three independent experiments. The statistical analysis was performed using the Student's unpaired two-tailed t-test (* $p < 0.05$; ** $p < 0.01$; *** $p < 0.001$).

A
Agonist
MB61^0
MB61^222
Fold increase
RIG-1
MDA5
TLR3
TLR7
TLR8
B
kDa
150
100
75
40
35
25
15
MB61^0
MB61^222
RIG-1
TLR3
TLR7
Optical Density (Arbitrary Units)
C
Fold increase
IRF3
NFkB
D
Optical Density (450 nm)
IRF3
pIRF3
NFkB
pNFkB
E
Fold increase
IL-1 α
IL-1 β
IL-4
IL-6
TNF-α
F
Not infected cells
pg/ml
G
H

**Figure 7. Proteomic analysis of Calu-3 cells infected with $MB61^{0}$ and $MB61^{222}$ isolates.** (A) Hierarchical Clustering of average protein lists from not infected Calu-3 cells and Calu-3 cells infected with SARS-CoV-2 $MB61^{0}$ or $MB61^{222}$ isolates, collected at 12, 24 and 48 h p.i.. The dendrogram was obtained by computing the average peptide spectrum matches of differentially expressed proteins selected by Linear Discriminant Analysis; Euclidean's distance metric and Ward 's methods were applied. Categories were reported in different colors accordingly to the isolates and time p.i.. (B) and (C) Histograms. Differentially expressed protein trend in the major pathways involved at 48 h p.i. comparing $MB61^{0}$ versus $MB61^{222}$ isolates. Orange bars and negative DAve values refer to upregulated proteins in $MB61^{222}$, while light blue bars and positive DAve values refer to upregulated proteins in $MB61^{0}$. For each protein, gene name and related DAve value (ratio of protein expression) are reported.

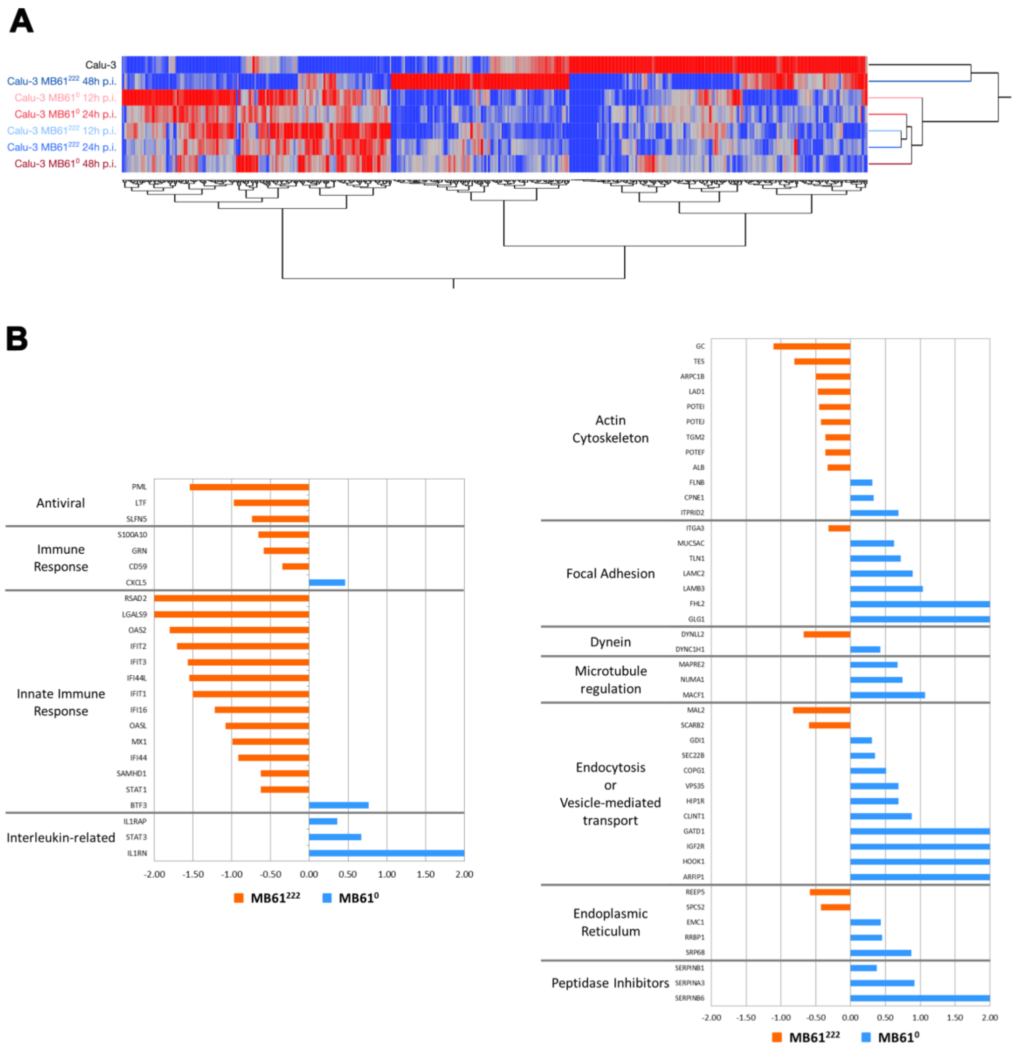

**Figure S1. Characteristics of iSNVs.**

Heatmap showing the percentage of paired-end mapped reads for synonymous and non-synonymous iSNVs accumulated throughout the viral genome over the course of long-term infection.

**Figure S2. Genetic characterization of longitudinally collected nasopharyngeal swabs**

(A) Location of the consensus mutations in the SARS-CoV-2 variants accumulated throughout the viral genome over the course of long-term infection. Consensus genome sequences were obtained for each sample by identifying, site by site, the most abundant nucleotide in the Illumina-derived aligned reads. Vertical dashes indicate synonymous (light blue) and non-synonymous (red) mutations; green dashes indicate a nucleotide substitution in the 5'-UTR non-coding region. Horizontal black dashes represent an amino acid deletion. (B) Representation of the dynamics of virus populations down to low frequencies during the entire period. Vertical axis represents the percentage (%) of the reads displaying mutations in the S gene.

**Figure S3. Growth kinetics of the $MB61^{0}$ and $MB61^{222}$ patient's isolates in Calu-3 and Caco-2 cell lines.** Human lung epithelial cell line Calu-3 (A) and Human Colorectal Adenocarcinoma cell line Caco-2 (B) cells were infected with $MB61^{0}$ and $MB61^{222}$ at a MOI of 0.01 alone or in combination (C). Supernatants from infected cells were harvested at designated time points for assessment of release of infectious viral particles by plaque assay performed on Vero E6 cells. Data shown are the mean and the standard error of the mean for three independent replicates. Student's unpaired two-tailed t-test was used to determine statistical significance for each time point (* $p < 0.05$, ** $p < 0.01$).

**Figure S4. Root Mean Square Deviation (RMSD) and Root Mean Square Fluctuation (RMSF).**

SARS-CoV-2 WT (black) and SARS-CoV-2 Mut (red) RBD. RMSD of CA atoms of RBD, RBM (residues 438-508), hACE2 and H1+H2+H18+β-turn (residues 19-101 + 319-365). RBM region of RBD is highlighted in grey as H1+H2 and H18+β-turn in hACE2 RMSF graph.

**Figure S5. Intermolecular hydrogen bonding frequency of hACE2 and RBD obtained from MD.**

**Figure S6. Protein-Protein Interaction (PPI) network of differentially expressed proteins in not infected and SARS-CoV-2-infected Calu-3 cells.** Not infected Calu-3 cells and Calu-3 cells infected with SARS-CoV-2 MB61$^{0}$ and MB61$^{222}$ strains were collected at 12, 24 and 48 h p.i.. Physical or/and functional interactions are highlighted by thicker edges and considering experimental (STRING score > 0.15) and database (STRING score > 0.35) annotated interactions. The networks were visualized by Cytoscape v.3.8.2 software, while biological processes were retrieved by BINGO Cytoscape's plugin. The color code of distinct nodes reflects the normalized aPSM value of a protein in each examined condition (up-regulated in red and down-regulated in blue light).

**Figure S7. Alignment and comparison among patient's sequences and VOCs in the N gene.** Genomic alignment of MB61$^{0}$ and MB61$^{222}$ isolates and five VOCs (Alpha, Beta, Gamma, Delta, Omicron). Nucleotide positions are referred to Wuhan sequence (Wuhan-Hu-1-NC_045512/MN908947.3), adopted as reference for this analysis. The TRS positions are highlighted by blue rectangle. Each nucleotide mutation is colored by type of change: start codon (yellow), noncoding (light blue), conservative (light green), synonymous (purple) and radical amino acid change (red).

## Supplementary Material

veac042_Supp

