# Supplementary material for: Competition for Dominance Within Replicating Quasispecies During Prolonged SARS-CoV-2 Infection in an Immunocompromised Host
Source: Virus Evol. 2022 May 21:veac042. doi: 10.1093/ve/veac042 (PMC9129230; doi:10.1093/ve/veac042)
Supplement: veac042_Supp [file veac042_supp.zip › supplementary.docx]

**Figure S1. Characteristics of iSNVs.**

Heatmap showing the percentage of paired-end mapped reads for synonymous and non-synonymous iSNVs accumulated throughout the viral genome over the course of long-term infection.

**Figure S2. Genetic characterization of longitudinally collected nasopharyngeal swabs**

(A) Location of the consensus mutations in the SARS-CoV-2 variants accumulated throughout the viral genome over the course of long-term infection. Consensus genome sequences were obtained for each sample by identifying, site by site, the most abundant nucleotide in the Illumina-derived aligned reads. Vertical dashes indicate synonymous (light blue) and non-synonymous (red) mutations; green dashes indicate a nucleotide substitution in the 5’-UTR non-coding region. Horizontal black dashes represent an amino acid deletion. (B) Representation of the dynamics of virus populations down to low frequencies during the entire period. Vertical axis represents the percentage (%) of the reads displaying mutations in the S gene.

**Figure S3. Growth kinetics of the MB61^0^ and MB61^222^ patient’s isolates in Calu-3 and Caco-2 cell lines.** Human lung epithelial cell line Calu-3 (A) and Human Colorectal Adenocarcinoma cell line Caco-2 (B) cells were infected with MB61^0^ and MB61^222^ at a MOI of 0.01 alone or in combination (C). Supernatants from infected cells were harvested at designated time points for assessment of release of infectious viral particles by plaque assay performed on Vero E6 cells. Data shown are the mean and the standard error of the mean for three independent replicates. Student’s unpaired two-tailed t-test was used to determine statistical significance for each time point (* p < 0.05, ** p < 0.01).

**Figure S4. Root Mean Square Deviation (RMSD) and Root Mean Square Fluctuation (RMSF).**

SARS-CoV-2 WT (black) and SARS-CoV-2 Mut (red) RBD. RMSD of CA atoms of RBD, RBM (residues 438-508), hACE2 and H1+H2+H18+β-turn (residues 19-101 + 319-365). RBM region of RBD is highlighted in grey as H1+H2 and H18+β-turn in hACE2 RMSF graph.

**Figure S5. Intermolecular hydrogen bonding frequency of hACE2 and RBD obtained from MD.**

**Figure S6.** **Protein-Protein Interaction (PPI) network of differentially expressed proteins in not infected and SARS-CoV-2-infected Calu-3 cells.** Not infected Calu-3 cells and Calu-3 cells infected with SARS-CoV-2 MB61^0^ and MB61^222^ strains were collected at 12, 24 and 48 h p.i.. Physical or/and functional interactions are highlighted by thicker edges and considering experimental (STRING score > 0.15) and database (STRING score > 0.35) annotated interactions. The networks were visualized by Cytoscape v.3.8.2 software, while biological processes were retrieved by BINGO Cytoscape’s plugin. The color code of distinct nodes reflects the normalized aPSM value of a protein in each examined condition (up-regulated in red and down-regulated in blue light).

**Figure S7. Alignment and comparison among patient’s sequences and VOCs in the N gene.**

Genomic alignment of MB61^0^ and MB61^222^ isolates and five VOCs (Alpha, Beta, Gamma, Delta, Omicron). Nucleotide positions are referred to Wuhan sequence (Wuhan-Hu-1-NC_045512/MN908947.3), adopted as reference for this analysis. The TRS positions are highlighted by blue rectangle. Each nucleotide mutation is colored by type of change: start codon (yellow), noncoding (light blue), conservative (light green), synonymous (purple) and radical amino acid change (red).
