# Supplementary figures and images for: Competition for Dominance Within Replicating Quasispecies During Prolonged SARS-CoV-2 Infection in an Immunocompromised Host
Source: Virus Evol. 2022 May 21:veac042. doi: 10.1093/ve/veac042 (PMC9129230; doi:10.1093/ve/veac042)

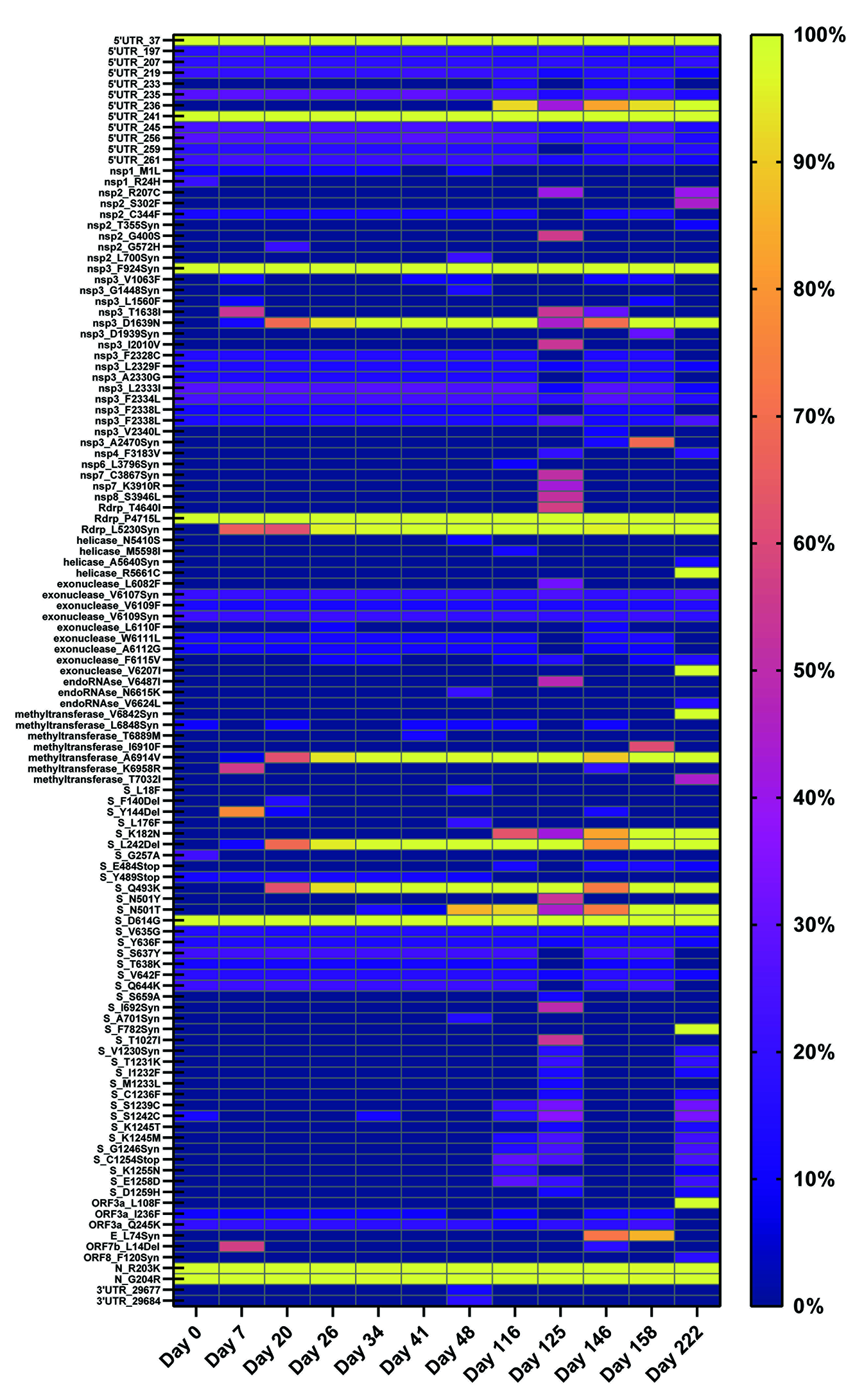

Supplement: veac042_Supp [file veac042_supp.zip › Figure S1.tif]

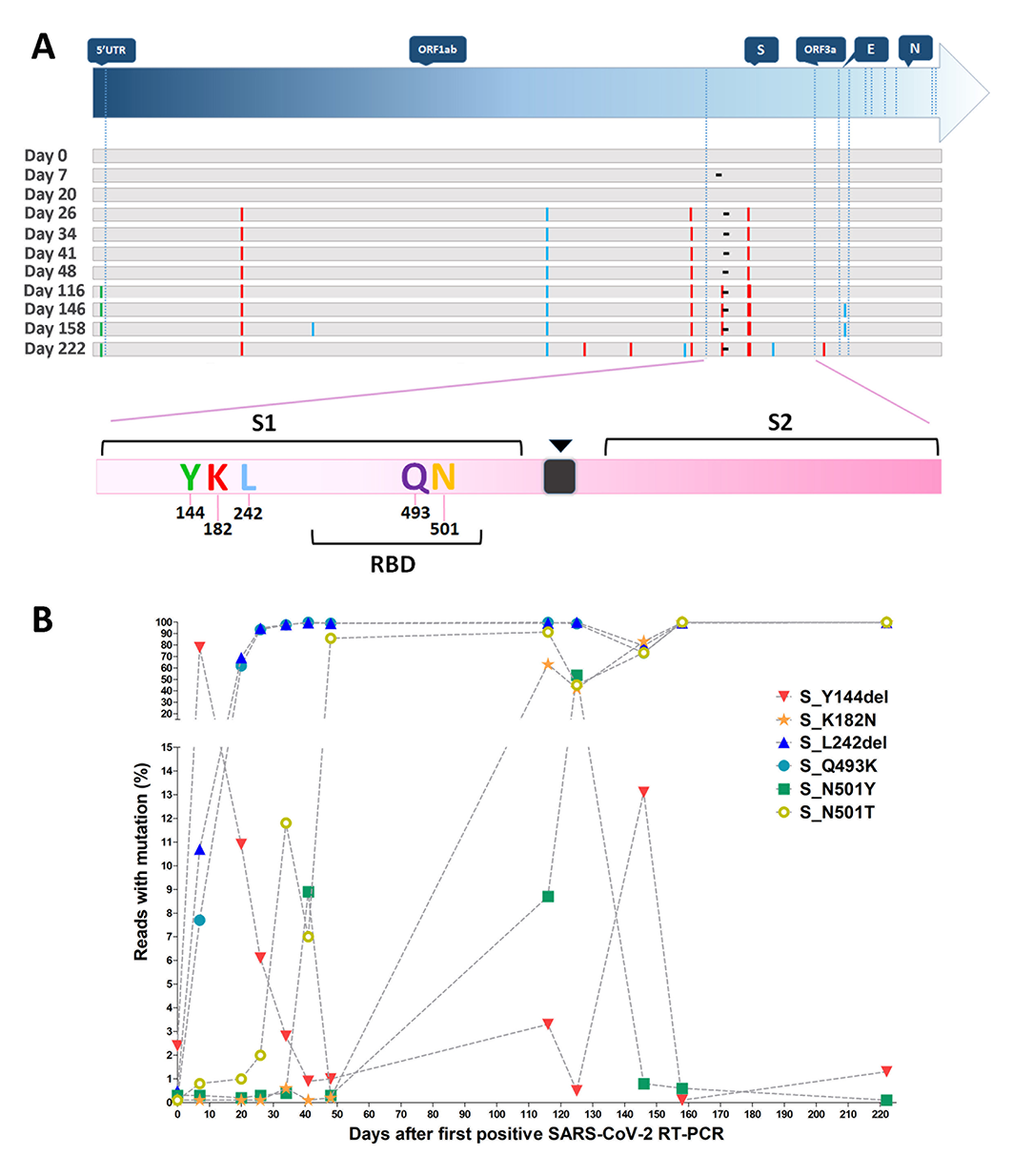

Supplement: veac042_Supp [file veac042_supp.zip › Figure S2.tif]

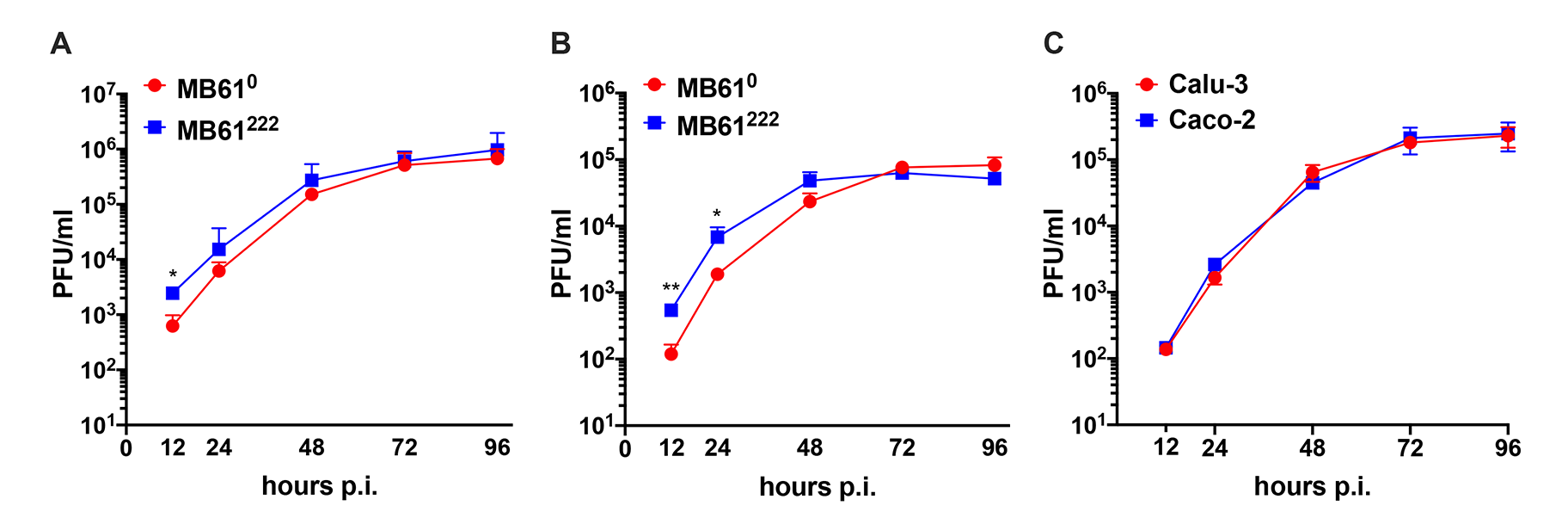

Supplement: veac042_Supp [file veac042_supp.zip › Figure S3 copia.tif]

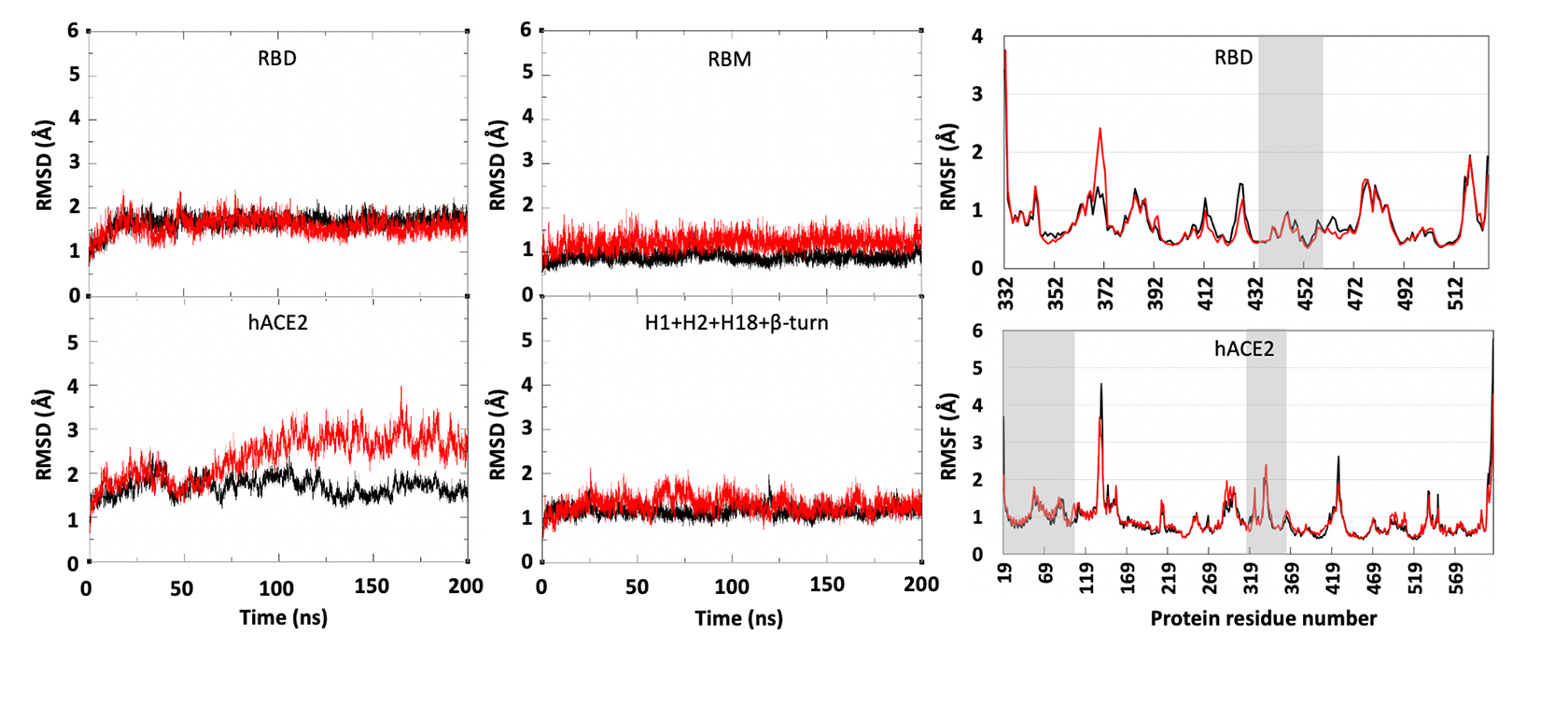

Supplement: veac042_Supp [file veac042_supp.zip › Figure S4 copia.tif]

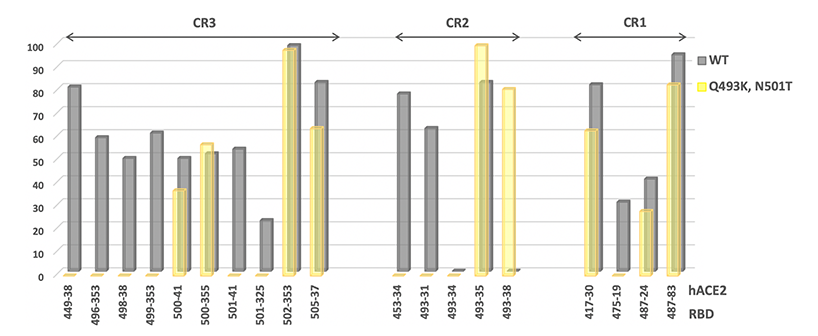

Supplement: veac042_Supp [file veac042_supp.zip › Figure S5 copia.tif]

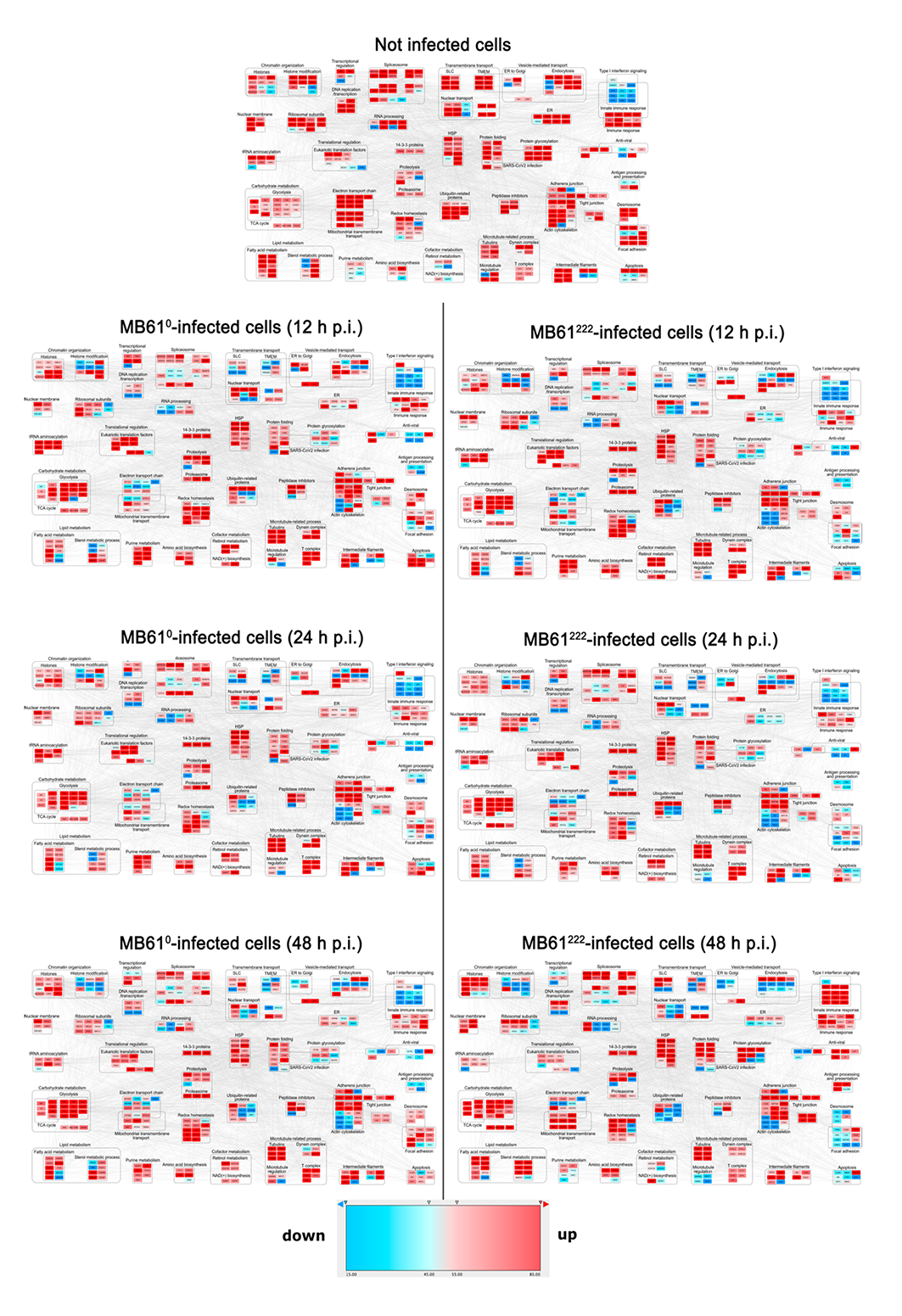

Supplement: veac042_Supp [file veac042_supp.zip › Figure S6.tif]

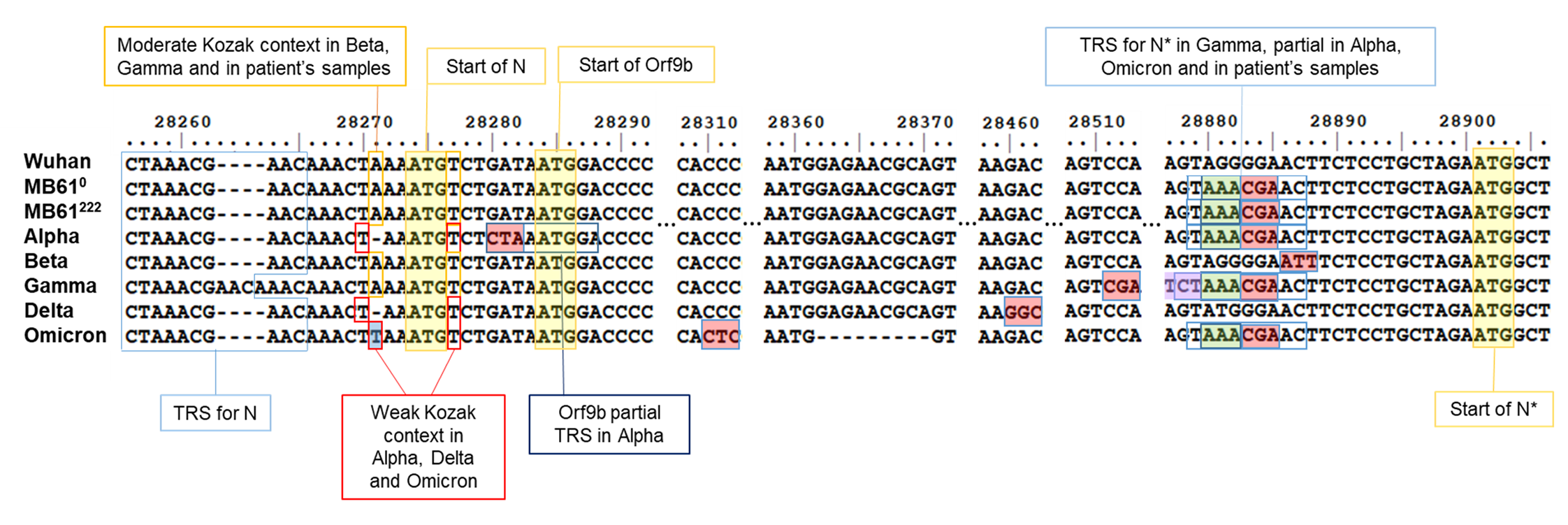

Supplement: veac042_Supp [file veac042_supp.zip › Figure S7.tif]
